# Block Copolymer-Enabled Low-Temperature Structural Battery Electrolytes Produced Using Polymerization-Induced Phase Separation

**DOI:** 10.1021/acsami.5c25105

**Published:** 2026-03-03

**Authors:** Sayyam Deshpande, Chen Wang, Coby Scrudder, Ramu Banavath, Jodie L. Lutkenhaus, Micah J. Green

**Affiliations:** a Artie McFerrin Department of Chemical Engineering, 14736 Texas A&M University, College Station, Texas 77843, United States; b Department of Materials Science and Engineering, 14736 Texas A&M University, College Station, Texas 77843, United States

**Keywords:** structural battery electrolyte, low temperature, low tortuosity, block copolymer, polymerization induced phase separation (PIPS), lithium-ion batteries, organic radical polymers

## Abstract

Structural battery electrolytes (SBEs) require both high ionic conductivity and high mechanical strength and stiffness. However, SBEs produced using the one-pot polymerization-induced phase separation (PIPS) synthesis method suffer from high tortuosity which decreases the effective ionic conductivity. Additionally, conventional liquid electrolytes demonstrate poor performance in low temperatures and are unsuitable for applications in cold climates. Here, we report SBEs generated using PIPS in the presence of an amphiphilic block copolymer (BCP), which modifies the solid–liquid interface that forms during polymerization, resulting in lower tortuosity and improved ionic conductivity. Using a low-temperature liquid electrolyte, the effect of BCP and resin content on the ionic conductivity and mechanical properties is examined. Only 1 wt % of BCP additive is needed to improve the ionic conductivities even at low temperature (2.34 × 10^–3^ S/cm at 25 °C and 1.28 × 10^–4^ S/cm at −30 °C, which are 78.3% and 99% higher than a similar SBE with no added BCP). The SBE, tested in both lithium iron phosphate and nitroxide radical polymer half-cells, demonstrates good compatibilities with discharge capacities of 145 mAh/g at a C-rate of 0.1 C and 103 mAh/g at a C-rate of 0.2 C, respectively, at 25 °C. At even lower temperatures of −20 °C, these cells retained 30 and 49% of their respective capacities.

## Graphical abstract

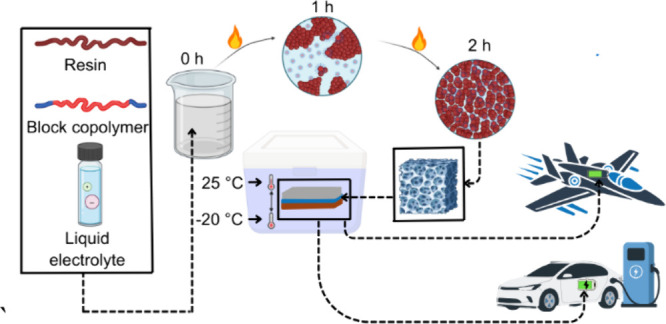

## 1 Introduction

Structural batteries comprised of multifunctional materials such as carbon fiber (CF) electrodes/current collectors and structural battery electrolytes (SBEs) can provide significant mass and volume savings in electric vehicles (EVs).^1–3^ For example, one report estimates that structural batteries could yield mass and volume savings of about 20% relative to an electric vehicle system using conventional batteries, leading to higher energy density and greater mileage.^3^ Due to their multifunctional character, structural batteries can safely bear mechanical loads and provide energy simultaneously.^3,4^ CFs are ideal scaffolds for structural battery applications because CFs possess a high Young’s modulus of 200–800 GPa and can reversibly intercalate lithium ions if used as an anode, similar to graphite.^5,6^ SBEs are multifunctional electrolytes that consist of a solid polymer phase that can function as a matrix to enable load transfer between CFs and a liquid electrolyte phase that provides ionic conductivity.^7–10^ SBEs are cured onto the CFs in situ to create structural batteries.^7^ SBEs are synthesized using reaction- or polymerization-induced phase separation (RIPS/PIPS), resulting in the formation of a percolating microstructure consisting of channels of liquid electrolyte in a polymer matrix.^8^ Due to the nature of PIPS, the orientation of the two phases is usually random with minimal control over the resulting microstructure.^11,12^ However, only a handful of studies have focused on SBEs at lower temperatures and with modified microstructure.

While solid polymer electrolytes (SPEs) such as polyethylene oxide (PEO) have been employed in solid-state batteries, they often possess poor interfacial adhesion with the electrodes,^13–17^ presenting a severe issue for structural applications. Typical Young’s moduli for SPEs containing semicrystalline PEO are between 1 and 70 MPa at 25 °C.^18–20^ Additionally, PEO-based SPEs possess low ionic conductivity (<10^–5^ S/cm) at ambient conditions, which decreases even further at low temperatures. In contrast to SPEs, SBEs contain a liquid phase that may retain higher conductivities.

SBEs are primarily synthesized using an in situ PIPS process; however, polymer blends and emulsion templating can also be used.^21–24^ During PIPS, resin (epoxy or vinyl monomer) and liquid electrolyte phase separate upon selective polymerization of the resin, resulting in the formation of a percolating, bicontinuous structure comprised of a solid polymer phase (load-bearing) and a liquid electrolyte phase (ionically conductive).^22,25^ SBEs synthesized using PIPS are more mechanically robust than most reported SPEs. For example, a bicontinuous electrolyte synthesized from equal amounts (by weight) of epoxy resin 5284 and a liquid electrolyte containing a mixture of LiTFSI/propylene carbonate (PC)/1-ethyl-3-methylimidazolium bis­(trifluoromethane­sulfonyl)­imide (EMIM-TFSI) had an ionic conductivity of 6.70 × 10^–4^ S/cm and Young’s modulus of 1.00 GPa at 25 °C.^9^

For a structural battery system, improving the mechanical properties and electrochemical properties are equally important. Unfortunately, SBEs synthesized by PIPS suffer a trade-off between mechanical properties and ionic conductivity based on the composition of the two phases,^8,11,26^ which is further exacerbated by the microstructure’s high and uncontrollable tortuosity, decreasing the SBE’s effective ionic conductivity.^27,28^ Bicontinuous SBEs reported in prior literature have high tortuosity (τ) values between 1.50 and 3 leading to hindered lithium ion transport.^27,28^ Ideally, a τ value close to 1 is desirable. The addition of 5 wt % of an amphiphilic BCP synthesized from glycidyl methacrylate (GMA) and quaternized (2-dimethylamino)­ethyl methacrylate (DMAEMA) to bisphenol A-diglycidyl ether (BADGE) and EMIM-TFSI followed by curing using PIPS possessed a Young’s modulus of 800 MPa and ionic conductivity of 0.28 mS cm^–1^ at 25 °C due to the decrease in the SBE’s feature sizes.^12^ That system was, however, not designed for low temperatures, and the effect of BCP content or SBE tortuosity was not explored. Another report showed that a styrene/ionic liquid based polymer electrolyte containing 45 wt % of block copolymer polyethylene oxide-polypropyleneoxide-polyethylene oxide (PEO-PPO-PPO) synthesized using PIPS demonstrated an ionic conductivity of 10^–3^ S/cm, an elastic modulus greater than 1 MPa at 70 °C, and a tortuosity factor of 5.9.^29^ Despite having high τ values, this study highlights that varying the amount of BCP can change tortuosity in the electrolytes. Another report synthesized a polymer electrolyte by PIPS using a PEO macromolecular chain transfer agent, styrene, divinyl benzene, and ionic liquid to obtain an ionic conductivity >10^–3^ S/cm and Young’s modulus of 1 GPa at 25 °C.^30^ They, however, did not evaluate the performance of their electrolytes in batteries or at low temperatures. Taken together, these studies indicate that BCPs can potentially reduce the feature sizes of SBEs, leading to improved ionic conductivities. However, a systematic understanding of BCP effects on structure and properties—especially at low temperature—is lacking.

Structural batteries may find application in extreme environments (<−60 °C) for EV applications in cold climates and aerospace.^31,32^ The average temperature on Mars is −60 °C, fluctuating between −80 °C on a summer night to −130 °C on a winter night and 30 °C during the day. The surface temperature of the moon varies from 130 °C during the day to −170 °C at night.^33,34^ Currently, commercial lithium ion batteries (LIBs) based on an ethylene carbonate (EC) electrolyte and graphite anode perform poorly at temperatures below 0 °C,^35^ thus presenting a challenge for their effective operation at subzero temperatures. This motivates the development of structural LIBs operable at low temperatures. Unfortunately, the trade-off between mechanical properties and ionic conductivity of SBEs becomes more pronounced at lower temperatures.^36^ Specifically, ion mobility in the liquid electrolyte becomes restricted as viscosity increases; at even lower temperatures, the electrolyte may even freeze, leading to a complete loss of conductivity.^37,38^ Maintaining a conductivity of at least 10^–4^ S/cm for lithium-ion battery (LIB) applications at low temperatures is necessary.^39^ SPEs operating at temperatures as low as −30 °C have been demonstrated previously.^40–42^ Specifically, an SPE containing poly­(vinyl ethylene carbonate) had a discharge capacity of 104 mAh/g at a temperature of −15 °C at 0.1 C in a lithium (Li)/lithium iron phosphate (LFP) cell configuration.^42^ However, none of these studies evaluated the mechanical properties of the SPE at even lower temperatures.

Previously, we designed an SBE that had an ionic conductivity of at least 10^–4^ S/cm and Young’s modulus of 32 MPa at −10 °C.^36^ The liquid electrolyte phase of that SBE was 1 M LiTFSI in fluoroethylene carbonate (FEC)/diglyme (1:9), which demonstrated excellent low temperature stability and did not freeze even at −90 °C. The SBE, however, demonstrated poor ionic conductivity (<10^–4^ S/cm) below −10 °C because of its high tortuosity (τ = 1.80). Other reports on SBEs operating at low temperatures are limited. For example, an SBE containing 2.3 M LiTFSI in ionic liquid and commercial epoxy MTM57 had an ionic conductivity on the order of 10^–4^ S/cm at −30 °C, and tortuosity was not evaluated.^11^ However, they did not evaluate the Young’s modulus of their SBE at low temperatures or test their SBE in a battery. Thus, the primary focus should be on improving the ionic conductivity of SBEs at low temperatures by reducing their tortuosity.

Here, we demonstrate an SBE (Figure 1) containing a cross-linked polymer network with minimal quantities of a triblock amphiphilic polyethylene glycol-polypropylene glycol-polyethylene glycol (PEG-PPG-PEG) BCP and a LiTFSI/diglyme/FEC electrolyte using PIPS. The chemical structures of each SBE component are shown in Figure 1. Diglyme and FEC impart low temperature stability to the liquid electrolyte.^36^ PEG-PPG-PEG was chosen because, during phase separation, the more polar PEG blocks interact with the liquid electrolyte, and the less polar PPG blocks interact with the vinyl monomer resin. The added BCP serves to lower the interfacial energy between the dissimilar phases, allowing for the formation of smaller domains during the curing process. We hypothesized that this mixed compatibility would lead to an SBE with lower tortuosity and finer morphological features. The resulting SBEs were characterized by using scanning electron microscopy (SEM), thermogravimetric analysis (TGA), tensile testing, differential scanning calorimetry (DSC), and battery testing in Li/LFP and Li/poly­(2,2,6,6-tetramethyl­piperidinyloxy methacrylate) (PTMA)-*co*-glycidyl methacrylate (GMA) cell configurations. Results indicate that the BCP does indeed produce lower tortuosity SBEs, resulting in higher conductivities and effective performance in both half cells. The multifunctional nature of these SBEs shows great promise for their use in low temperature structural battery applications.

**Figure 1:**
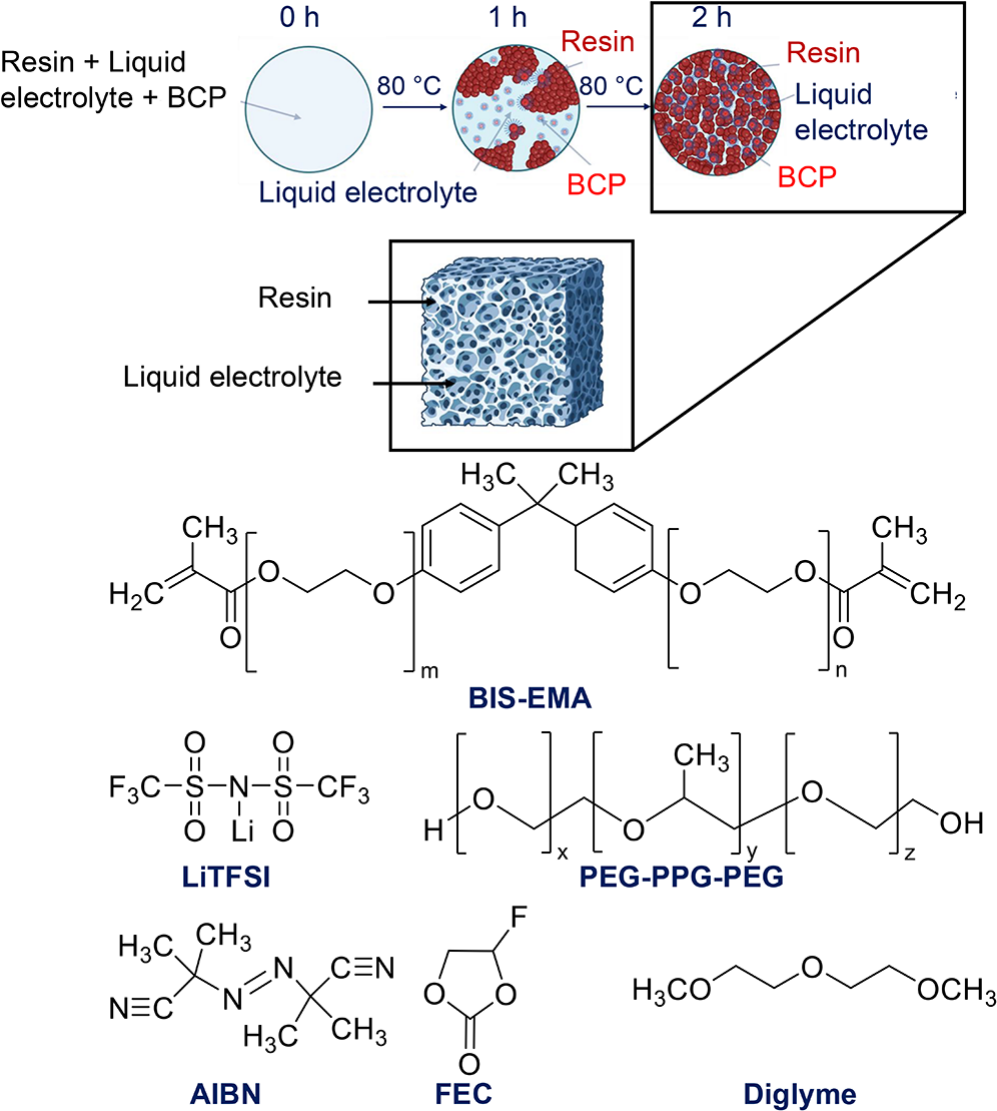
Schematic of block copolymer (BCP)-enhanced structural battery electrolytes (SBEs) and chemical structures of the compounds used to synthesize them. The liquid electrolyte is 1 M LiTFSI in FEC/diglyme (1:9 m/m).

## 2 Experimental Section

### 2.1 Materials

LiTFSI salt (99.99% trace metals basis and anhydrous), diglyme (99.9% pure and anhydrous), azobisisobutyronitrile (AIBN) (98% pure), FEC (99% pure, <200 ppm acid, anhydrous), 1-methyl 2-pyrrolidinone (NMP, 99.5% pure and anhydrous), m-chloroperoxybenzoic acid (m-CPBA), and triblock copolymer PEG-PPG-PPG, (10 wt% PEG, average M_n_: 1100 g/mol, quality level 100) were procured from Sigma-Aldrich. Bisphenol A ethoxylated dimethacrylate (Bis-EMA) (m + n = approximately 4, stabilized with HQ, molecular weight: 540.65 g/mol) resin was procured from TCI Chemicals. Stainless steel spacers with a thickness of 0.2 mm (EQ-CR20-Spacer-02), stainless steel springs (EQ-CR20WS), stainless-steel casings (EQ-CR2032-CASE, SS-316), aluminum foil, polyvinylidene fluoride (PVDF, molecular weight: 600 000 g/mol, quality level 100), LFP powder (>97% pure), and lithium chips with a diameter of 16 mm and thickness of 0.6 mm (EQ-Lib-LiC) were procured from MTI corporation. Super P carbon black (≥99% pure, metals basis) and isopropyl alcohol (IPA, > 99% pure) were purchased from VWR. Release agent (Chem Trend Chemlease EZ 45-90) was procured from Chem Trend. 2,2,6,6-tetramethyl-4-piperidinyl methacrylate (TMPM) was purchased from Tokyo Chemical Industry Co., Ltd. GMA was purchased from MTI corporation. All chemicals and reagents were used as received.

### 2.2 SBE Preparation

LiTFSi salt was added to a mixture of 90 wt % diglyme and 10 wt % FEC under constant stirring until the concentration of the resulting electrolyte was 1M. The electrolyte was then mixed with different weight ratios of BCP and Bis-EMA (along with 1 wt % AIBN in the resin) in a planetary mixer (Thinky AR-100) for 10 min at a speed of 2000 rpm. The obtained mixture was pipetted into a mold which was assembled by separating two glass slides with a 1 mm thick silicon spacer. Before assembling the mold, each glass slide was bath sonicated in IPA for 20 min and dried in an oven at 80 °C for 20 min. The mixture was then cured in the mold at 80 °C for two h on a hot plate inside an argon-filled glovebox (<0.1 ppm of O_2_, <0.1 ppm of H_2_O). The resulting SBEs were then punched into 16 mm discs using a die-cutter and assembled into a coin cell using a coin cell assembly procedure for electrochemical testing. For tensile testing, the mixtures were pipetted into dog-bone shaped molds and cured outside the glovebox in an oven at 80 °C for 2 h. This process may allow for moisture uptake, thus leading to an under-estimation of the mechanical properties.

### 2.3 Electrochemical Impedance Spectroscopy (EIS)

EIS measurements were performed on the SBE samples and battery cells in a frequency range of 10 mHz to 1 MHz at an amplitude of 10 mV using a potentiostat (GAMRY reference 600). Each EIS measurement was carried out at open circuit potential (OCP). In cells for measuring the ionic conductivity of the SBEs, the spacers functioned as blocking electrodes. EIS measurements were carried out on the SBEs at different temperatures (25 °C, 10 °C, 0 °C, −10 °C, −20 °C, −30 °C, and −40 °C) in an environmental chamber (TENNEY ENVIRONMENTAL). Each cell was equilibrated at the desired temperature for 10 min before testing. The ionic conductivities were calculated using eq 1.

1 $$\sigma =\frac{l}{RA}$$

where R is the resistance of the SBE calculated from the x-intercept of the linear region of the Nyquist plot, A is the cross-sectional area of the spacer (201 mm^2^), and l is the thickness of the SBE. The resistance of the SBE was obtained by subtracting the measured resistance from EIS and the short circuit resistance (1.38 Ω) of a blank cell containing spacers and a spring.

### 2.4 Tensile Testing

Samples for mechanical testing were prepared using the same compositions described above. These mixtures were poured into a type V dog-bone shaped polydimethylsiloxane (PDMS) mold. The mixtures in the mold were then cured in an oven outside the glovebox at 80 °C for 2 h. Tensile properties were evaluated using the MTS Insight Electromechanical Testing System equipped with 2.5 kN and 30 kN load cells. The testing system utilized wedge-type grips with a fixed bottom grip and a movable top grip. All tensile tests were conducted according to ASTM Standard D638. For low temperature measurements, the samples were enclosed in a cryogenic environmental chamber from Thermcraft and equilibrated at the desired temperatures (10 °C, 0 °C, −10 °C, −20 °C, −30 °C, and −40 °C) for 20 min before testing. The 80 and 90 wt % electrolyte stress–strain curves were smoothed using the first order Savitzky–Golay function. Extensometers were not used during this test, and percent elongation values were calculated as a function of crosshead displacement divided by gauge length. At low tensile loads (<0.5 kN), the deflection of the machine is significantly lower than the sample deflection, and the machine compliance is negligible. Young’s modulus was calculated from the slope of the linear region of the stress–strain curve.

### 2.5 DSC Measurements

DSC was conducted on the glovebox-cured 30 wt % resin, 1 wt % BCP SBE sample using a TA Instruments DSC Q200. The sample was first quenched to −50 °C at a rate of 10 °C/min and held isothermally at −50 °C for 5 min. The sample was then ramped up from −50 to 30 °C at a rate of 2 °C/min and held isothermally at 30 °C for 3 min. DSC was also conducted on a 100 wt % electrolyte sample. The sample was first quenched to −90 °C at a rate of 10 °C/min and held isothermally at −90 °C for 5 min. The sample was then ramped up from −90 to 30 °C at a rate of 2 °C/min and held isothermally at 30 °C for 5 min.

### 2.6 ImageJ Analysis

To quantify the pore size of the SBE samples, ImageJ analysis was performed on their SEM images. The scale was first set to the appropriate value by equating pixels to the corresponding value in microns. Then a rectangular element was selected over the SEM image and cropped. Following this, straight lines were drawn on individual pores, and their diameters were measured. This was repeated 50 times, and the pore diameter was plotted as a histogram.

### 2.7 Liquid Electrolyte Extraction Procedure

Liquid electrolyte was extracted from the SBEs using an ethanol exchange process. The SBEs were soaked in ethanol in vials for 10 min followed by bath sonication for 30 min. The vials were then drained of the liquid, and the same procedure was repeated five times. After this procedure, the SBE samples were heated in a vacuum oven at 60 °C for 24 h. The weight of the SBE samples was measured before and after the ethanol swap. Macroscopic shrinkage was not observed in the samples.

### 2.8 SEM Imaging of Samples

Liquid electrolyte from each broken dog-bone sample after mechanical testing was extracted using the liquid electrolyte extraction procedure mentioned above. The cross-section of the samples was imaged using a scanning electron microscope (FEI Quanta 600 FE-SEM) and (JEOL JSM-7500F) at accelerating voltages between 5 and 20 kV. Each sample was sputter coated with a 10 nm thick layer of Pt/Pd before imaging.

### 2.9 TGA

To evaluate the effectiveness of the liquid electrolyte extraction procedure, TGA was performed on the glovebox-cured 30 wt % resin, 1 wt % BCP SBE sample, and a 100 wt % resin sample using a TA Instruments TGA 5500. 3–5 mg of sample was heated in a platinum pan from 25 to 1000 °C under a nitrogen environment with a ramp rate of 20 °C/min.

### 2.10 Density Calculations for the SBE

The liquid electrolyte was first extracted from the BCP containing SBEs using the liquid electrolyte extraction procedure mentioned above. The mass of the BCP containing SBEs was measured before and after electrolyte extraction. The density of resin in the BCP containing SBEs was calculated by dividing the mass of the SBE postelectrolyte extraction by its dimensions.

### 2.11 PTMA-*co*-GMA Preparation

PTMA-*co*-GMA was synthesized using a procedure as previously described.^43^ TMPM (5 g, 22 mmol) and 1% GMA (29 mL, 0.22 mmol) were dissolved in 10 mL of toluene, followed by the addition of AIBN (0.11 g, 0.67 mmol) to initiate the free-radical polymerization. The mixture was then heated to 60 °C for 48 h to allow the reaction to complete. After the reaction was completed, the mixture was washed with ethanol and dried under vacuum for 12 h to give PTMPM-*co*-GMA. Then PTMPM-*co*-GMA (1 g, 4.4 mmol based on TMPM monomer) and m-CPBA (2 equiv, 1.5 g, <77%) were dissolved in 10 mL dicloromethane (DCM) and reacted at 25 °C for 3 h. The mixture was then washed with water and 0.5 M sodium bicarbonate solution. The orange colored organic phase was separated from the aqueous phase using a separatory funnel. Then, hexane (20 equiv, v/v) was added to the organic phase to precipitate the solid. The solid was then isolated by using vacuum filtration and dried at 50 °C for 24 h to obtain orange colored PTMA-*co*-GMA powder. The Mn = 18000 g/mol, Mw = 42200 g/mol, and dispersity (D) = 2.28 of the PTMA-*co*-GMA was determined using gel permeation chromatography (GPC). The radical content was also previously determined using electronic paramagnetic resonance (EPR) spectroscopy.^43^

### 2.12 Cyclic Voltammetry (CV)

A slurry comprising LFP (80 wt %), super P (10 wt %), and PVDF (10 wt %) in NMP was doctor-bladed onto aluminum foil. The electrodes were dried for 24 h at 25 °C, 1 atm pressure, and were then heated at 120 °C for 12 h under vacuum before cutting into 12 mm diameter circular discs for coin cell assembly. The LFP active material loading in these electrodes for all CV tests was 1.10–1.75 mg/cm^2^. Similarly, a slurry containing PTMA-*co*-GMA (50 wt %), super P (40 wt %), and PVDF (10 wt %) in NMP was used to prepare PTMA-*co*-GMA electrodes on aluminum foil. The PTMA-*co*-GMA electrodes were dried at room temperature for 12 h under vacuum and heated to 175 °C for 3 h for cross-linking. The electrodes and coin-cells were prepared similarly to the LFP electrodes. The LFP active material loading in these electrodes for all CV tests was 1.05–1.15 mg/cm^2^. CV was performed on SBEs in a half-cell configuration with LFP or PTMA-*co*-GMA as the cathode, lithium chip as the anode, and SBE as both the electrolyte and the separator in CR-2032 coin cells. The lithium chips were punched into 12 mm diameter circles before their usage as an anode. 30 μL liquid electrolyte was added to improve the wettability of the SBE with the electrodes. The coin cells were rested for 24 h and CV was then performed in a voltage window of 2.5–4.2 V vs Li/Li^+^ for Li/LFP cells and 3–4.1 V vs Li/Li^+^ for Li/PTMA-*co*-GMA cells using a potentiostat (GAMRY reference 600) at a scan rate of 0.1 mV/s. The same procedure mentioned above was used to prepare the SBEs, except a 250 μm thick silicon spacer was used to separate the glass slides instead. The glass slides were dipped in release agent and heated in an oven at 100 °C for 10 min before being used to cure the SBEs. A digital image of the mold is shown in Figure S1.

### 2.13 Lithium Plating and Stripping Test Procedure

For lithium plating and stripping, the SBEs were assembled into symmetric coin cells with 12 mm diameter lithium metal electrodes on each side. The cells were then cycled at current densities of 0.5 mA/cm^2^ and 1.0 mA/cm^2^ for 1 h per charge and discharge. The voltage was recorded as a function of time.

### 2.14 Galvanostatic Charge–Discharge (GCD) Measurements

Cell conditioning for GCD was done by running CV for three cycles using the procedure mentioned above. GCD testing was carried out on the Li/LFP coin cells between 2.5–4.1 V vs Li/Li^+^ and 3–4.1 V vs Li/Li^+^ for Li/PTMA-*co*-GMA cells using a battery tester (Arbin Instruments) at C-rates of 0.1 C, 0.2 C, 0.5 C, and 1 C for Li/LFP cells and C-rates of 0.2 C, 0.5 C, and 1 C for Li/PTMA-*co*-GMA cells for 5 cycles at each C-rate to evaluate the performance of the SBE in a battery cell charge–discharge. The same procedure was conducted at temperatures of 25 °C, 10 °C, 0 °C, −10 °C, and −20 °C to evaluate the low-temperature charge performance of the cells. Each cell was equilibrated at the temperatures mentioned above for 1 h before testing. Long-term cycling was performed at 0.2 C and 25 °C for Li/LFP cells and at 0.5 C and −10 °C for Li/PTMA-*co*-GMA cells.

## Results and Discussion

Different amounts of BCP (1 wt %, 2.5 wt %, and 5 wt %) were combined in a mixture containing 30 wt % resin and balance liquid electrolyte before curing. Ideally, a higher resin-to-liquid electrolyte ratio is preferred; however, for lithium-ion batteries to operate, a minimum ionic conductivity of 10^–4^ S/cm is desired.^23^ To achieve ionic conductivities above 10^–4^ S/cm at low temperatures, the resin content of the SBEs was fixed at 30 wt %. During curing and PIPS, resin and liquid electrolyte phase separate upon selective polymerization of the resin, resulting in the formation of a percolating, bicontinuous structure comprised of a solid polymer phase (load-bearing) and a liquid electrolyte phase (ionically conductive).^22,25^ The added BCP lowers the surface energy between the two phases through self-assembly at the interface between the polymer phase and the liquid electrolyte phase during PIPS. The final BCP-containing SBEs appear opaque (Figure S2). Further increasing the resin content to 60 wt % results in SBEs that appear translucent, consistent with other reports;^44^ unfortunately, the conductivity of this sample is prohibitively low for reasonable application.

First, we investigated the effect of varying BCP content on the ionic conductivity of the SBEs containing a fixed resin content (30 wt %) at 25 °C. No separator was used in the cells. Increasing the BCP content in the SBE enhances the ionic conductivity of the 30 wt % resin SBE as shown in Figure 2. A leftward shift in the Nyquist plots shown in Figure 2a shows that the presence of BCP decreases the SBE’s impedance. Increasing the BCP content in the SBEs from 0 wt % to 5 wt % increases the ionic conductivity from 3.01 × 10^–4^ S/cm to 2.34 × 10^–3^ S/cm **(** Figure 2b**)** – a 7.3-fold improvement. The mechanical properties of the SBEs were then evaluated as a function of BCP content. The addition of any amount of BCP softens the polymer matrix. This can be observed in Figure 2c where the Young’s modulus of the 30 wt % resin SBE reduces from 74.9 to 28.6 MPa on increasing BCP content from 0 wt % to 5 wt %. The corresponding stress–strain curves are shown in Figure 2c. This reduction in Young’s modulus could be specific to the block copolymer chosen in this study. The block copolymer chosen (PEG-PPG-PEG) has two more-polar (PEG) blocks per the less-polar PPG block. The finer morphology (see below) afforded by the presence of the BCP leads to the altered mechanical properties. The effect of BCP content on the ultimate tensile stress (UTS) and strain to failure of the SBEs is shown in Table S1. Specifically, the UTS decreases from 5.15 MPa to 2.02 MPa upon increasing BCP content from 0 wt % to 5 wt %. The strain to failure of the SBEs increases from 0.124 to 0.245 upon increasing BCP content from 0 wt % to 5 wt % (Table S1). This result demonstrates the tunability of both the mechanical properties and the conductivity by tuning the BCP content. The ideal properties of the SBE may depend on the specific deployment of the structural battery, requiring a certain amount of tunability. For example, an SBE to be utilized in the environs of a carbon fiber reinforced laminate should have a high modulus. For an SBE to be utilized in an application where some amount of strain is expected, as in flexible devices, a high strain-to-failure is also preferred.

**Figure 2:**
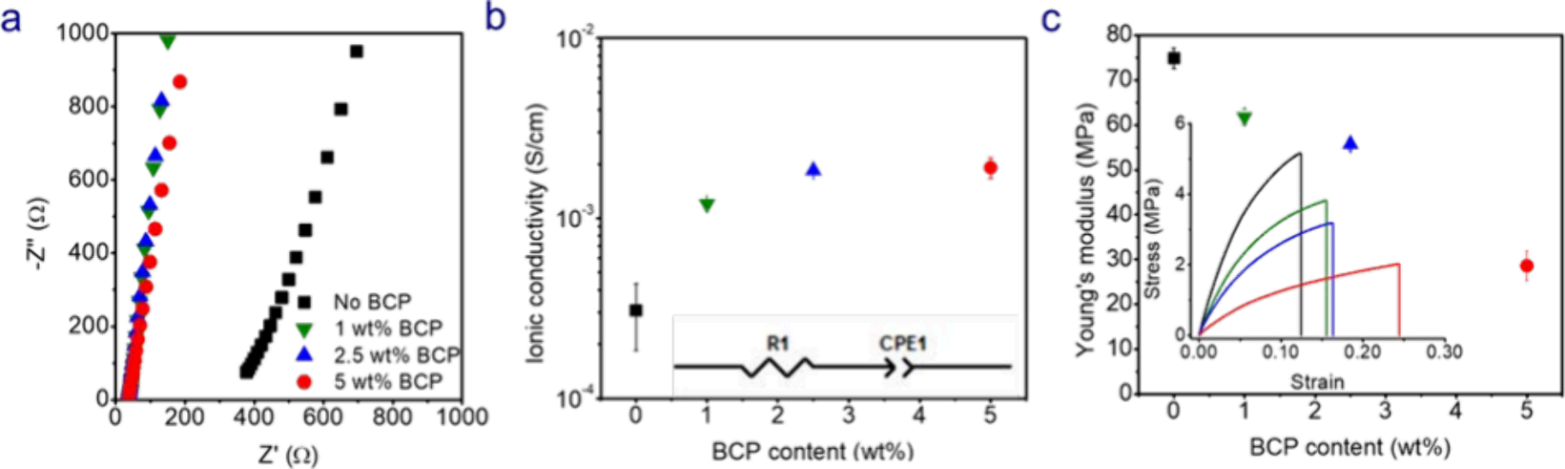
(a) Nyquist plots, (b) ionic conductivities, and (c) Young’s moduli of SBEs containing 30 wt % resin as a function of BCP content at 25 °C. Liquid electrolyte makes up the balance. Note: Error bars are present but sometimes small.

To verify the occurrence of PIPS and observe the resultant morphologies, SEM was performed on the cross-section of the SBEs from which the liquid electrolyte had been removed. Figure S3 shows TGA experiments that evaluate the effectiveness of the electrolyte extraction process. The microscopy images revealed the formation of a porous, bicontinuous structure with vacant channels that the liquid electrolyte had occupied and small, interconnected resin particles **(** Figures 3a and 3c**)**. It is noteworthy that, compared to the SEM of the SBEs without any BCP **(** Figures 3b and 3d**)**,^36^ the BCP-containing samples appear to have smaller and more interconnected pores even at higher resin contents (up to 50 wt % resin). The porosity of the SBEs was calculated using eq 2 below.

2 $$\varepsilon =\frac{m_{sample}-m_{resin}}{m_{sample}}$$

where m_sample_ is the mass of the SBE before electrolyte extraction and m_resin_ is mass of the SBE after electrolyte extraction.

**Figure 3:**
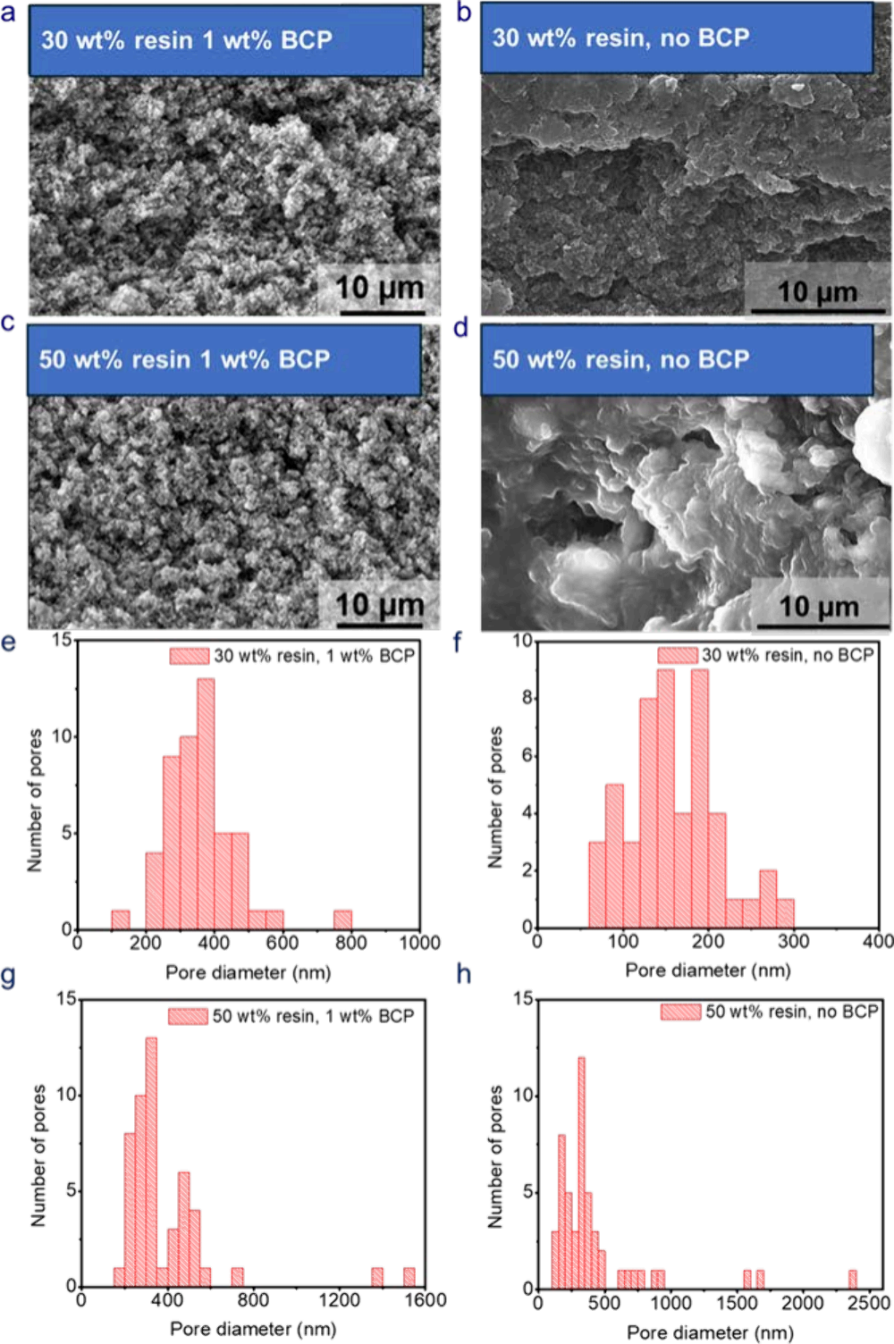
SEM images of SBEs containing (a) 30 wt % resin and 1 wt % BCP, (b) 30 wt % resin and 0 wt % BCP, (c) 50 wt % resin and 1 wt % BCP, (d) 50 wt % resin and 0 wt % BCP, and (e–h) respective pore size distribution estimated using ImageJ analysis.

A comparison of the porosities and densities of the BCP-containing SBEs and those without BCP is presented in Table S2 and Table S3, respectively. It is observed that the addition of BCP increases the porosity of the SBEs. This agrees with the SEM images that show that the BCP-containing SBEs appear more porous at high resin contents (>50 wt % resin) compared to their BCP-free counterparts. For example, the addition of 1 wt % of BCP to the 30 wt % resin SBE increases the porosity from 0.413 to 0.560. One would expect the porosities of the two samples to be largely the same, but these results indicate otherwise. This is because eq 2 relies on the efficacy of the liquid extraction from the porous matrix. If there are isolated pores, as is likely the case for the sample without BCP, then liquid within those pores cannot be extracted, thus leading to a lower apparent porosity. Therefore, the difference can be attributed to the BCP generated more connected pores.

The pore size distribution of the SBE samples was determined using ImageJ analysis on the corresponding SEM images as shown in Figure 3e–3h. The BCP produces microstructures with larger pore sizes and smaller resin particle sizes. For example, the 30 wt % sample shows an average pore size of 359 nm whereas the corresponding sample without BCP shows an average pore size of 157 nm. Digital images of SBE dogbones show that samples transform from being opaque to clear as the resin content is increased (as the pore size decreases), Figure S2.

The ionic conductivities of the BCP-containing SBEs and those without BCPs were calculated as a function of temperature using EIS. The x-intercepts of the Nyquist plots shown in Figure S4 were used to calculate the resistance. The Nyquist plots for the full frequency range are shown in Figure S5. The corresponding Bode plots at 25 °C are shown in Figures S6–S9. A rightward shift in the Nyquist plots can be observed with increasing resin content and decreasing temperature. This can be attributed to higher resistance to lithium-ion diffusion through the SBE caused by a higher solid content and a decrease in the diffusion coefficient of Li^+^ at low temperatures, respectively. The ionic conductivities of the BCP-containing SBEs were plotted as a function of temperature at fixed resin contents (Figure 4a**)**. For a given resin content, ionic conductivity decreases with lower temperatures as expected. Conversely, ionic conductivity decreases with increasing resin content at a fixed temperature which is also expected. Also, DSC conducted on both the 1 wt % BCP SBE sample (Figure S10a **)** containing 30 wt % resin and the neat liquid electrolyte (Figure S10b) showed that neither exhibited freezing even down to −90 °C. Higher resin contents were attempted, but the ionic conductivity for the 60 wt % resin sample with 1 wt % BCP was found to be too resistive (<10^–4^ S/cm) even at 25 °C.

**Figure 4:**
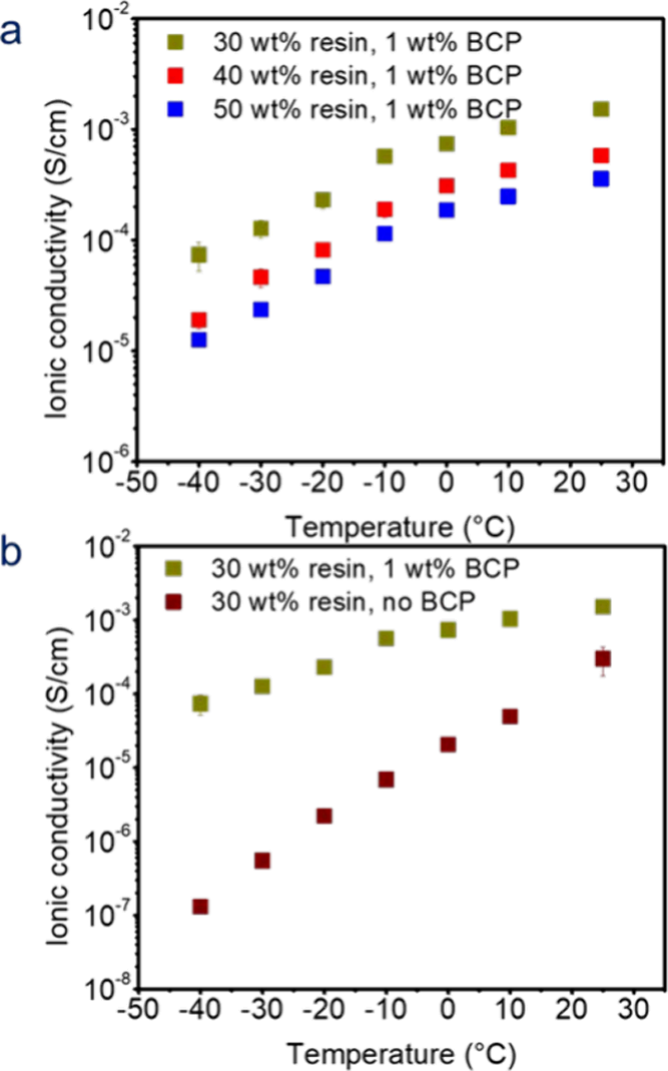
Ionic conductivities of (a) 1 wt % BCP SBEs with different resin contents as a function of temperature and (b) 1 wt % BCP and 0 wt % BCP SBEs with 30 wt % resin as a function of temperature. Note: Error bars are present but sometimes small.

A comparison of the ionic conductivities of 1 wt % BCP SBEs and no-BCP SBEs at a fixed resin content of 30 wt % is shown in Figure 4b to reveal the effect of the added BCP. The BCP-containing SBEs show higher ionic conductivity than their non-BCP counterparts at the same resin content and temperature. For example, at 25 °C and 30 wt % resin, the addition of 1 wt % of BCP to the SBE improves ionic conductivity from 3.01 × 10^–4^ S/cm to 1.52 × 10^–3^ S/cm – a 5-fold improvement. Relative to the neat electrolyte, Figure S10a,b, the ionic conductivity of the 1 wt % BCP SBE with 30 wt % resin was reduced by 58% at room temperature, which is expected due to the presence of SBE’s solid phase.

To examine the temperature effects, the linear region of ionic conductivity data was fit using an Arrhenius relationship with temperature (Figure S11, Table S4). Liquid-like activation energies (29.9–35.9 kJ/mol for the SBEs and 28.4 kJ/mol for the neat liquid electrolyte) confirm that ionic conductivity occurs within the liquid phase of the SBE. The ionic conductivity of SBE with added BCP exhibited a lower activation energy than the one without, which may be attributed to the higher porosity and lower tortuosity afforded by the BCP’s addition. The tortuosity factor (τ) of the SBEs was calculated using eq 3:

3 $$\tau =\frac{\kappa_{0}\cdot \varepsilon }{\kappa_{SBE}}$$

where κ_0_ is the ionic conductivity of the liquid electrolyte at 25 °C and κ_SBE_ is the ionic conductivity of the SBE sample at 25 °C. For example, the addition of 1 wt % of BCP to the 30 wt % resin SBE decreases its tortuosity factor from 17.5 to 1.33 (Tables S5 and S6).

The mechanical properties of the SBEs containing 1 wt % BCP were evaluated using tensile testing, Figure S13 and Figure S14. The UTS values of the SBEs increased with increased resin content at 25 °C, as expected, Figure 5a. The UTS also increased with decreasing temperature, exhibiting a maximum at – 30 °C for the SBE containing 30 wt % of resin and 1 wt % of BCP **(** Figure 5b**)**; the decrease in UTS at −40 °C may be attributed to the brittleness of the SBE at low temperatures. The Young’s moduli of the SBEs containing 1 wt % BCP increased linearly with increased resin content, Figure 5c. As resin content increases, the stiffer phase contributes more prominently to load-transfer. For the 30 wt % resin and 1 wt % BCP SBE in Figure 5d, the Young’s modulus increased from 61.8 to 727 MPa as the temperature decreased from 25 °C to – 40 °C. The specific Young’s modulus was also calculated for the SBEs by considering the SBE’s density after electrolyte extraction in which similar trends with resin content and temperature were obtained, Figure S15. The 1 wt % BCP SBE with 60 wt % resin yielded the highest specific modulus of 230 MPa·cm^3^/g. The specific modulus of the 1 wt % BCP SBE with 30 wt % resin increased from 117 MPa·cm^3^/g to 1380 MPa·cm^3^/g as temperature decreased from 25 °C to −40 °C.

**Figure 5:**
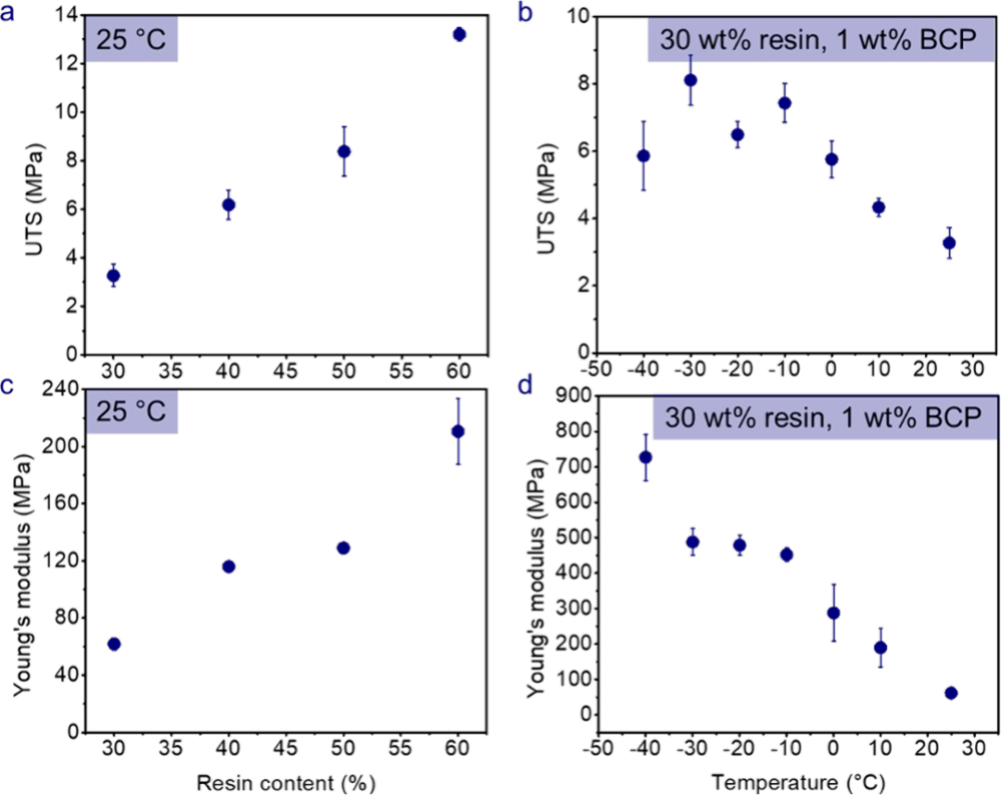
Mechanical properties of SBEs at different resin contents and temperatures. UTS of (a) 1 wt % BCP at 25 °C at different resin contents and of (b) 30 wt % resin SBE as a function of temperature. Similarly, Young’s modulus of (c) 1 wt % BCP SBEs at 25 °C and of (d) 30 wt % resin SBE.

In Figure 6, we compare the trade-off between Young’s modulus and ionic conductivity of our SBEs with a BCP (gray region) and those without.^9,36,45–50^ The modulus of neat resin at 25 °C and ionic conductivity of neat liquid electrolyte at 25 °C represent the respective maxima of the two properties that can be achieved at 25 °C. At 25 °C, the SBE containing 1 wt % of BCP and 30 wt % of resin demonstrated Young’s modulus of 61.8 MPa and ionic conductivity of 1.52 × 10^–3^ S/cm; at −30 °C, that same SBE demonstrated an increased Young’s modulus of 488 MPa and a decreased ionic conductivity of 1.27 × 10^–4^ S/cm. For structural applications, a Young’s modulus of 70 MPa at 25 °C is not sufficient, however, our modulus at low temperatures is comparable to previously reported SBE moduli at 25 °C.^21,23^ Stiffer electrolytes tend to possess lower ionic conductivities than their nonstiffer counterparts.^21,23^ Those reported electrolytes possess ionic conductivities slightly above the minimum ionic conductivity (10^–4^ S/cm) for LIBs to operate at ambient conditions but those SBEs were examined at lower temperatures.^23^ The performance of our SBEs at low temperatures is comparable to the performance of many SBEs at 25 °C reported in the literature. More specifically, our SBE system was compared against bicontinuous electrolytes comprised of BADGE/EMIM-TFSI,^45^ PEG-epoxy,^46^ 1-butyl-3-methyl-imidizolium-tetrafluoroborate (BMIBF_4_)/lithium tetrafluoroborate (LiBF_4_)-epoxy,^47^ EMIM-TFSI/LiTFSI-epoxy,^9,48^ 1-butyl-3-methylimidazolium­bis­(trifluoromethyl­sulfonyl)­imide (BMIM-TFSI)/LiTFSI-BADGE,^49^ and LiTFSI-succinonitrile/PEO.^50^ However, these examples did not examine low-temperature performance. Taken together, our SBE shows an effective combination of stiffness and conductivity at low temperatures.

**Figure 6:**
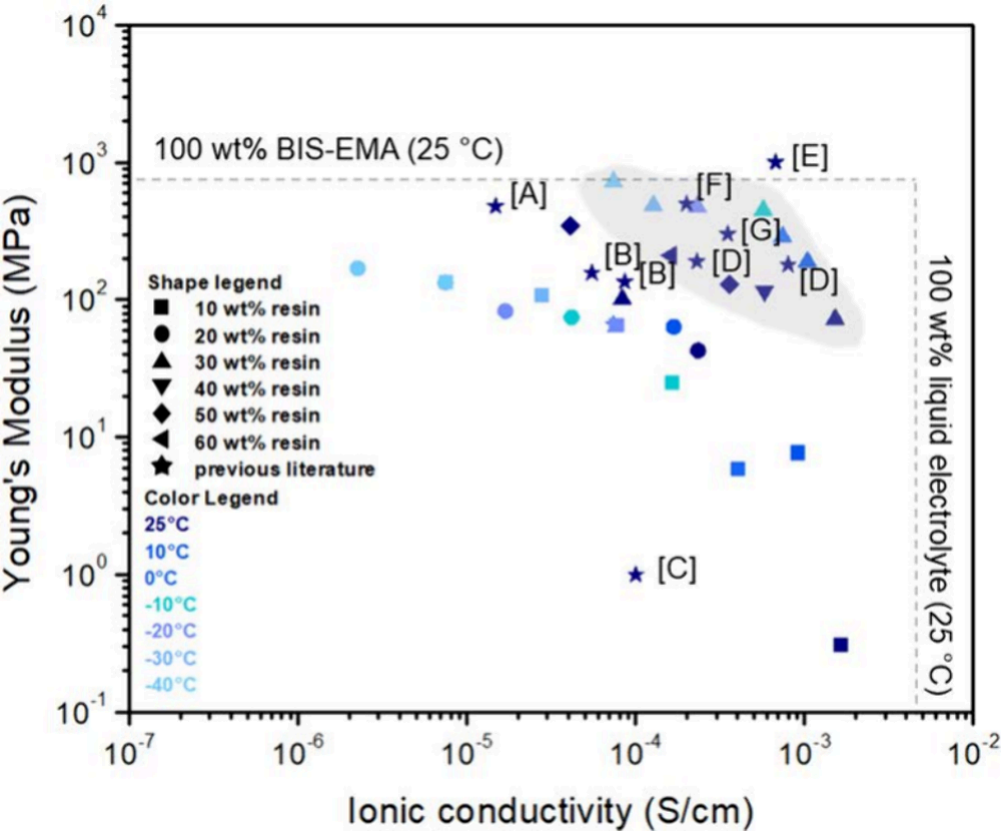
Ashby plot of Young’s modulus vs ionic conductivity for SBEs. The dashed lines indicate the properties of the neat resin and electrolyte. The new data reported here for the 1 wt % BCP containing SBEs are shown in the gray-shaded region. Our previously reported data are also shown, all outside the gray region.^36^ The data show the trade-off between the modulus and ionic conductivity. Stars indicate representative SBEs for batteries and supercapacitors from the literature corresponding to A,^45^ B,^46^ C,^47^ D,^48^ E,^9^ F,^49^ and G.^50^ For comparison, a Celgard separator with liquid electrolyte 1 M LiPF_6_ in ethylene carbonate (EC)/diethyl carbonate (DEC) has a Young’s modulus of 500 MPa ^51^ and ionic conductivity of 2.4 × 10^–3^ S/cm at 25 °C.^52^

To examine the performance of the SBE in a Li/LFP cell, CV (Figure 7a) was conducted for a cell with the 30 wt % resin SBE containing 1 wt % BCP at a scan rate of 0.1 mV/s. The CV shows a pair of distinctive peaks (E_1/2_ = 3.43 V and ΔE = 0.36 V), indicating that the SBE allows for ion conduction to both electrodes. Further, because no large excursions in voltage were observed, the SBE in the tested voltage range of 2.5 to 4.2 V exhibited acceptable stability. To further assess stability, lithium plating and stripping was performed on the 1 wt % BCP, 30 wt % resin SBE in a Li–Li symmetric cell at different current densities (0.5 and 1 mA/cm^2^). The SBE demonstrated stability at low current densities (0.5 mA/cm^2^ and 1 mA/cm^2^), signified by the decrease and stabilization of overpotential with time (Figure 7b). Next, EIS was performed on the Li/LFP cells before cycling (after formation) and an Li|SBE|Li cell after 100 cycles at 0.5 mA/cm^2^ (Figure 7c). The low-frequency semicircle observed in the EIS spectrum of Li/LFP cells was attributed to the SEI resistance (R_SEI_) at the Li metal surface, and the high-frequency semicircle represented the charge transfer resistance (R_ct_) associated with the redox reactions at the electrode surfaces. The EIS response also shows that the LFP interface had significantly higher resistance than the Li metal interface for the SBE cells. In the future, curing the SBE directly on the LFP electrodes could potentially alleviate this issue. Also, the EIS response of a Li|SBE|Li cell showed a low-frequency semicircle, signifying the formation of an SEI. The rate capability of the Li/LFP cells was also evaluated for the SBE at different C-rates (0.1, 0.15, and 0.2 C) for five cycles each (Figure 7d). The SBE showed high discharge capacities of 142 mAh/g at 25 °C and a C-rate of 0.1 C. After 70 cycles at 0.2 C-rate, the SBE-based cell retained 90.7% of its original capacity (Figure 7e**)**. Taken together, these results show that the SBE performs well in an inorganic electrode battery system. We next examined the effect of temperature on the rate capability of the Li/LFP cells containing the SBE (Figure 7f, Table S7). The cells displayed high discharge capacities of 145–147 mAh/g at 25 °C at a C rate of 0.1 C, with discharge capacity decreasing with increasing C-rate, as expected. At lower temperatures, the capacity decreased due to the slower Li^+^ diffusion and lower ionic conductivity of the SBEs. However, the cells still showed appreciable discharge capacity at 0.1 C at low temperatures (42–43 mAh/g at −20 °C). Upon returning to a C-rate of 0.1, each cell recovered almost 100% of their original capacity. The low temperature C-rate capability for the Li/LFP cell was better than that of a Li/Graphite cell reported for an SBE system without BCP.^36^ The Li/LFP cells demonstrate a discharge capacity of 42–43 mAh/g while the Li/Graphite cells failed to deliver any discharge capacity at −10 °C.^36^ The failure of the Li/Graphite cells can be attributed to lithium plating on graphite which is accelerated at low temperatures.^53,54^ Our results show that SBEs can be integrated into Li-ion batteries at temperatures as low as −20 °C.

**Figure 7:**
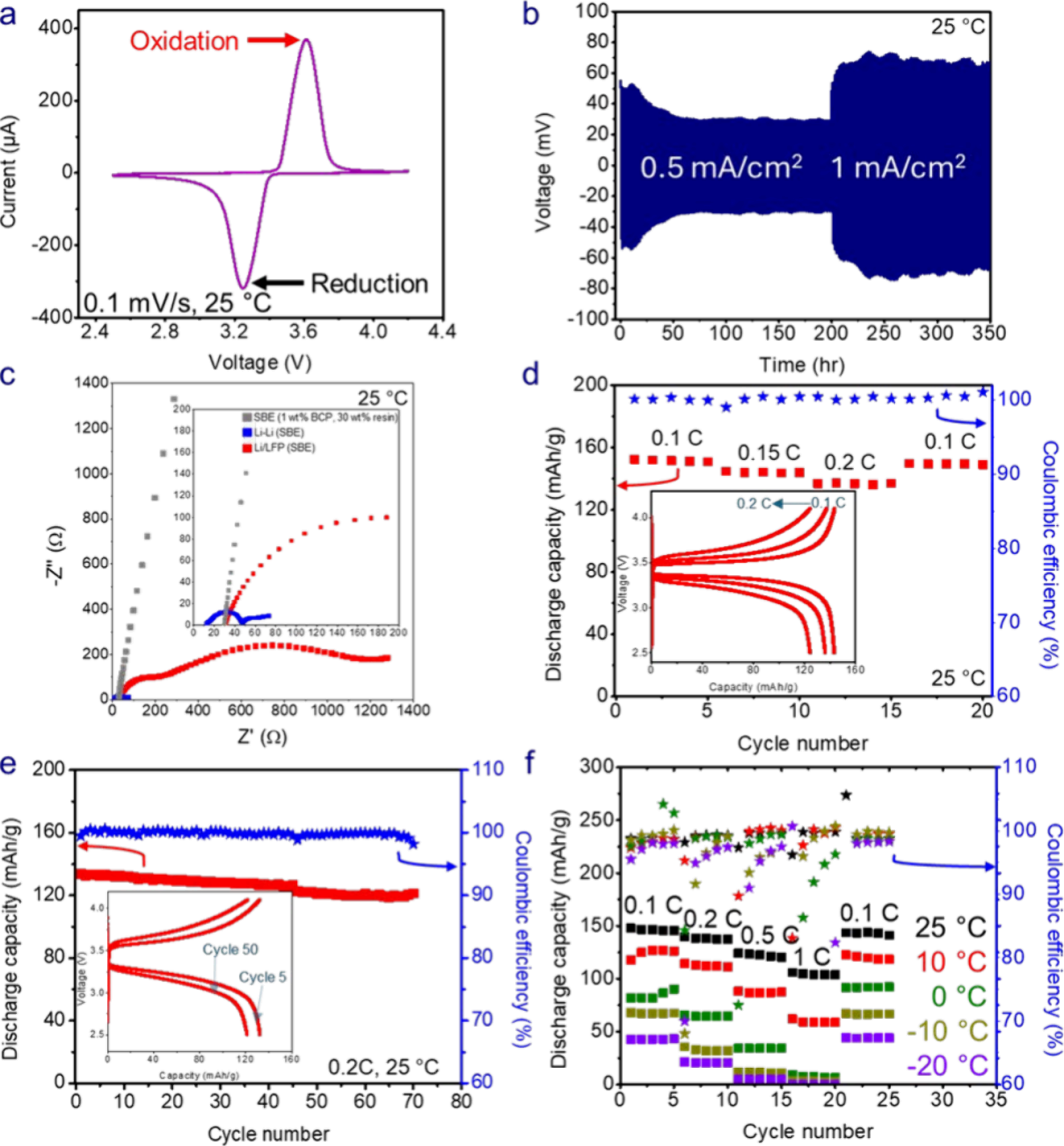
(a) Cyclic voltammetry (CV) after formation (before cycling) of Li/LFP cells at 25 °C and a scan rate of 0.1 mV/s, (b) lithium plating and stripping at different current densities for symmetric lithium cells at 25 °C, (c) EIS response after formation (before cycling) at 25 °C for Li/LFP cells and after 100 cycles at 0.5 mAh/cm^2^ for the Li–Li cell, (d) rate capability at 25 °C and different C-rates (0.1 C, 0.15 C, 0.2 C) for Li/LFP cells, (e) long-term cycling data for Li/LFP cells at 0.2 C and 25 °C, and (f) low-temperature rate capability for Li/LFP cells with 1 wt % BCP, 30 wt % resin SBE as electrolyte and separator (stars represent Coulombic efficiency).

The SBE was next examined in a Li/PTMA-*co*-GMA half-cell in which PTMA-*co*-GMA is a redox-active polymer that stores charge through the reversible redox reaction of the nitroxide radical and oxoammonium cation (C_theo_ of PTMA-*co*-GMA = 110 mAh/g). The CV in Figure 8a shows a pair of distinctive peaks (E_1/2_ = 3.7 V and ΔE = 0.2 V), indicating that the SBE allows is compatible with the PTMA-*co*-GMA electrode. No large excursions in voltage were observed, and the cells were stable in the tested voltage range of 3 to 4.1 V. Next, EIS was performed before cycling (after formation) at different temperatures (25 °C, 10 °C, 0 °C, −10 °C, and −20 °C) (Figure 8b). The EIS response shows that the R_ct_ increases at lower temperatures due to slower reaction kinetics. Also, the R_s_ values of the cells do not increase significantly at lower temperatures. We next evaluated the effect of temperature on the rate capability of the Li/PTMA-*co*-GMA cells containing the SBE (Figure 8c, Table S7). The cells displayed high discharge capacities of 102–103 mAh/g and 89–91 mAh/g at C-rates of 0.2 and 0.5 C at 25 °C, respectively. At lower temperatures, the capacity decreased as expected; however, the cells still showed appreciable discharge capacity at 0.2 C at low temperatures (48–50 mAh/g at −20 °C). The Coulombic efficiency of the cells improved at lower temperatures indicating a lower occurrence of side reactions.^43^ Additionally, the cells showed negligible capacity loss at 0.2 C from 10 °C to −10 °C. After 100 cycles at 0.5 C-rate at −10 °C, the SBE-based cell retained nearly 100% of its original capacity and showed only a slight decrease in R_ct_ (Figure 8d).

**Figure 8:**
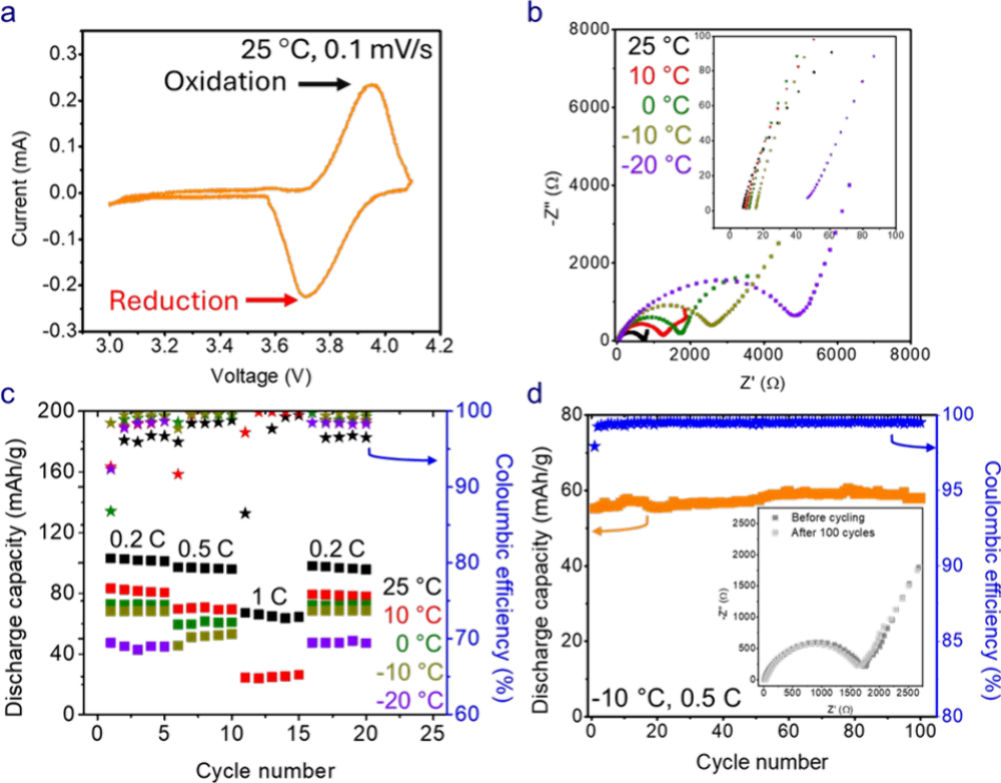
(a) Cyclic voltammetry (CV) after formation (before cycling) of Li/PTMA-*co*-GMA cells at 25 °C and a scan rate of 0.1 mV/s. (b) EIS response after formation (before cycling) at 25 °C, 10 °C, 0 °C, −10 °C, and −20 °C for Li/PTMA-*co*-GMA cells. (c) Rate capability at 25 °C, 10 °C, 0 °C, −10 °C, and −20 °C and different C-rates (0.2, 0.5, and 1 C) for Li/PTMA-*co*-GMA cells. (d) Long-term cycling data for Li/PTMA-*co*-GMA cells at 0.5 C and −10 °C. Stars represent Coulombic efficiency.

To compare the specific power and specific energy of the Li/LFP cells and Li/PTMA-*co*-GMA cells, a Ragone plot (Figure S16, Table S8 **)** was constructed. The Li/LFP cell demonstrated slightly higher specific energy (399 Wh/kg) and specific power (246 W/kg) than the Li/PTMA-*co*-GMA cell (350 Wh/kg and 203 W/kg respectively) at 25 °C and a C-rate of 0.5 C. This result is expected due to LFP’s higher theoretical capacity than PTMA-*co*-GMA. However, at lower temperatures, the PTMA-*co*-GMA cell outperformed the LFP cell in terms of specific energy and demonstrated comparable power. For example, at −10 °C and a C-rate of 0.5 C, PTMA-*co*-GMA delivered higher specific energy (196 Wh/kg) and specific power (204 W/kg) compared to LFP, which otherwise failed to deliver significant discharge capacity under the same conditions and thus, negligible energy. This can be attributed to the fast conversion-based mechanism of PTMA-*co*-GMA, which is unaffected by sluggish desolvation of lithium ions that LFP otherwise suffers at low temperatures. Our results show that the SBEs can demonstrate compatibility and low-temperature operation up to −20 °C with both organic and inorganic electrodes. Taken together, the Ragone plot shows that the SBE can yield respectable energy and power in lithium half cells for both LIBs and organic batteries.

## Conclusions

Structural batteries are promising for their potential to act as multifunctional components in automative and aerospace applications, storing energy while simultaneously bearing mechanical loads. Whereas most structural batteries have been designed for room temperature applications, lower temperature operation in arctic or space environments presents an extreme challenge due to hindered ion transport in the electrolyte. Here, we have evaluated a structural battery electrolyte designed for low temperature operation. We demonstrated that the addition of a block copolymer to a resin-electrolyte mixture produced structural battery electrolytes of lower tortuosity and higher conductivity than those without the block copolymer. During PIPS, the BCP lowered the interfacial tension between the liquid and cured phases, leading to more connected and less tortuous channels of liquid electrolyte inside a thermoset polymer matrix. The polymer matrix phase provided structural rigidity while the liquid electrolyte phase facilitated high ionic conductivity. Adding any amount of BCP softened the SBEs. The tortuosity factor for SBEs containing 30 wt % resin decreased from 17.5 to 1.33 and the effective porosity increased from 0.413 to 0.560 with the addition of 1 wt % BCP. The SBEs containing 30 wt % resin and 1 wt % BCP at −30 °C exhibited a relatively high ionic conductivity of 1.28 × 10^–4^ S/cm and Young’s modulus of 488 MPa. The SBE demonstrated good compatibility in both LIB and organic battery half-cells at temperatures as low as −20 °C.

Future work should focus on improving the ionic conductivity of the SBEs and cell design so that lower temperature (<−50 °C) operability can be achieved. For example, curing the SBE directly on the electrodes would help reduce interfacial resistance, reducing polarization. Also, modifying the liquid electrolyte formulation for lower temperature operation is another promising approach. Last, the molecular weight of the BCP and its chemistry should be varied towards improving the mechanical properties of the SBEs. Overall, this work provides an in-depth understanding of the influence of BCPs on the morphology and properties of SBEs, as well as the low-temperature performance.

## Supplementary Material



## Data Availability

The data underlying this study are openly available in FigShare at https://figshare.com/s/00ee13cc916108493dfa.

## Notes

Digital images of mold for SBE preparation, digital images of dogbone samples, TGA data, UTS and strain to failure values of SBEs, SBE porosities, SBE tortuosity values, bode plots, Nyquist plots, Arrhenius equation, fits and fit parameters, DSC data, raw tensile stress–strain curves, SBE densities, ionic conductivity of neat liquid electrolyte, specific modulus data, discharge capacity values of SBE cells at different temperatures, Ragone plots, energy density and power density values of SBE cells at different temperatures (PDF)
