# Supplementary material for: Block Copolymer-Enabled Low-Temperature Structural Battery Electrolytes Produced Using Polymerization-Induced Phase Separation
Source: ACS Appl Mater Interfaces. 2026 Mar 3;18(10):15599–609. doi: 10.1021/acsami.5c25105 (PMC13006949; doi:10.1021/acsami.5c25105)
Supplement: Supplementary file 1 [file am5c25105_si_001.pdf]

SUPPORTING INFORMATION

Block copolymer-enabled low-temperature structural battery electrolytes produced using polymerization-induced phase separation

Sayyam Deshpande[a], Chen Wang[a], Coby Scrudder[a], Ramu Banavath[a], Jodie L. Lutkenhaus[a,b*], and Micah J. Green[a,b*]

Affiliations:

[a]*Artie McFerrin Department of Chemical Engineering, Texas A&M University, College Station, Texas 77843, USA*

[b]*Department of Material Science and Engineering, Texas A&M University, College Station, Texas 77843, USA*

[c]*Texas A&M Transportation Institute, Texas A&M University, College Station, Texas 77843, USA*

***Corresponding author's email: micah.green@tamu.edu, jodie.lutkenhaus@tamu.edu**

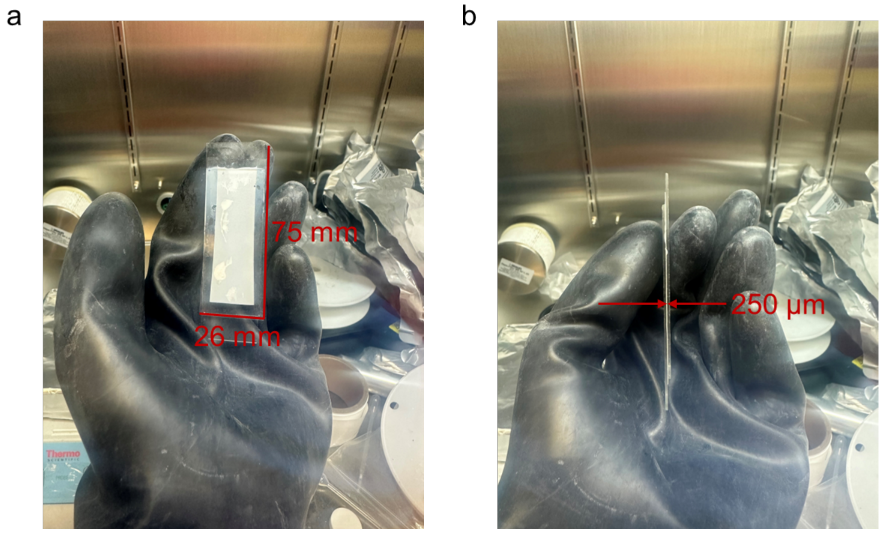

Figure S1. Digital images of mold used to cure the SBE for battery tests: a) top-down and b) side view.

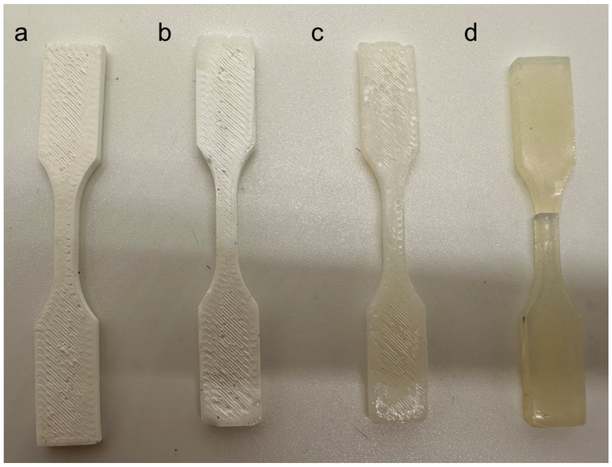

Figure S2. Digital images of 1 wt% BCP dogbone samples containing a) 30 wt% resin, b) 40 wt% resin, c) 50 wt% resin, and d) 60 wt% resin. The dimensions of all the samples are approximately 63.5 mm x 9.53 mm x 3.20 mm.

Table S1. UTS and strain of SBEs as a function of BCP content at 25 °C.

BCP content (wt%)	Ultimate tensite strength, UTS (MPa)	Strain
0	5.15	0.124
1	3.82	0.155
2.5	3.16	0.163
5	2.02	0.245

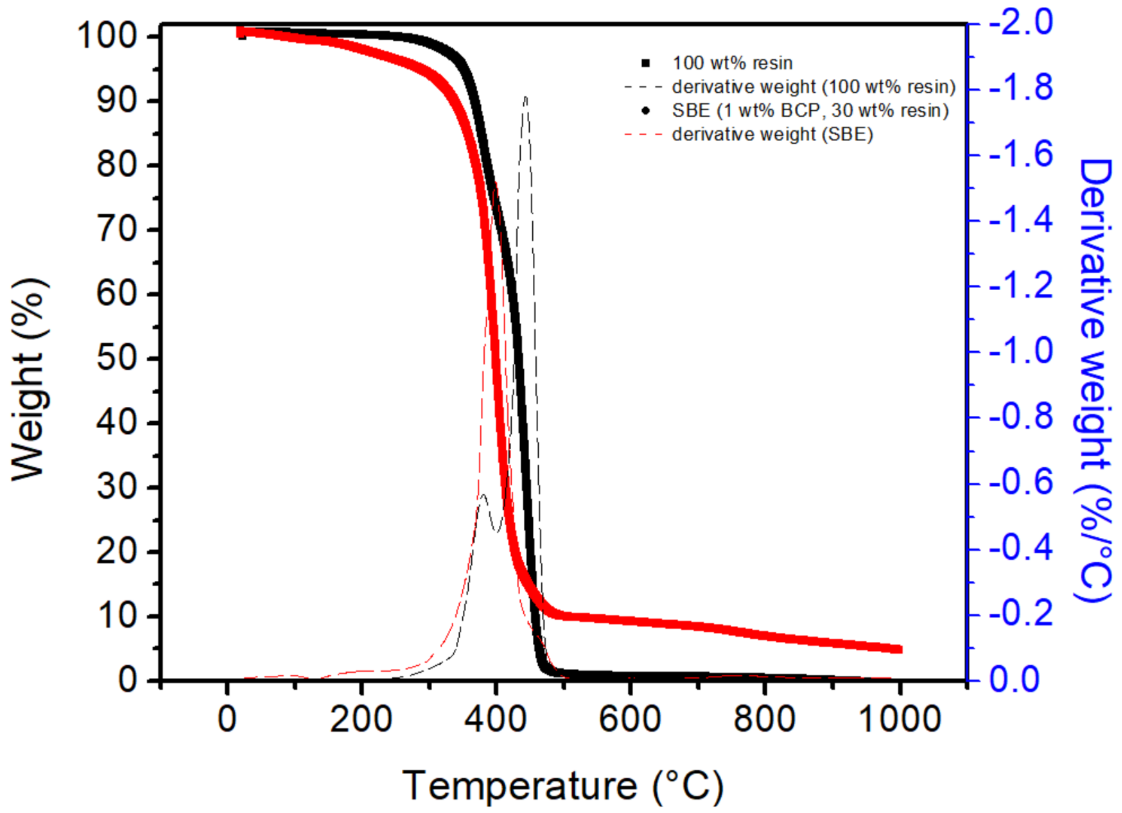

Figure S3. Thermogravimetric analysis (TGA) in nitrogen for the 100 wt% resin and SBE containing 1 wt% BCP and 30 wt% resin. Data between 200-350 °C shows ~6 wt% of unextracted liquid electrolyte after the ethanol extraction procedure. The remaining ~10 wt % of the SBE can be attributed to the char of the unextractable liquid electrolyte.[1] The neat (100 wt%) resin degrades completely at 450 °C.

Table S2. Porosity and post-extraction density of 1 wt% BCP containing SBEs.

Resin content (wt%)	Effective porosity (ε)	Density (g/cm^3)
30	0.560	1.20
40	0.385	1.15
50	0.297	1.19
60	0.149	1.08

Table S3. Porosity and post-extraction density of SBEs containing no BCP[2].

Resin content (wt%)	Effective porosity (ε)	Density (g/cm^3)
10	0.823	0.34
20	0.569	0.68
30	0.413	0.84
50	0.372	1.26

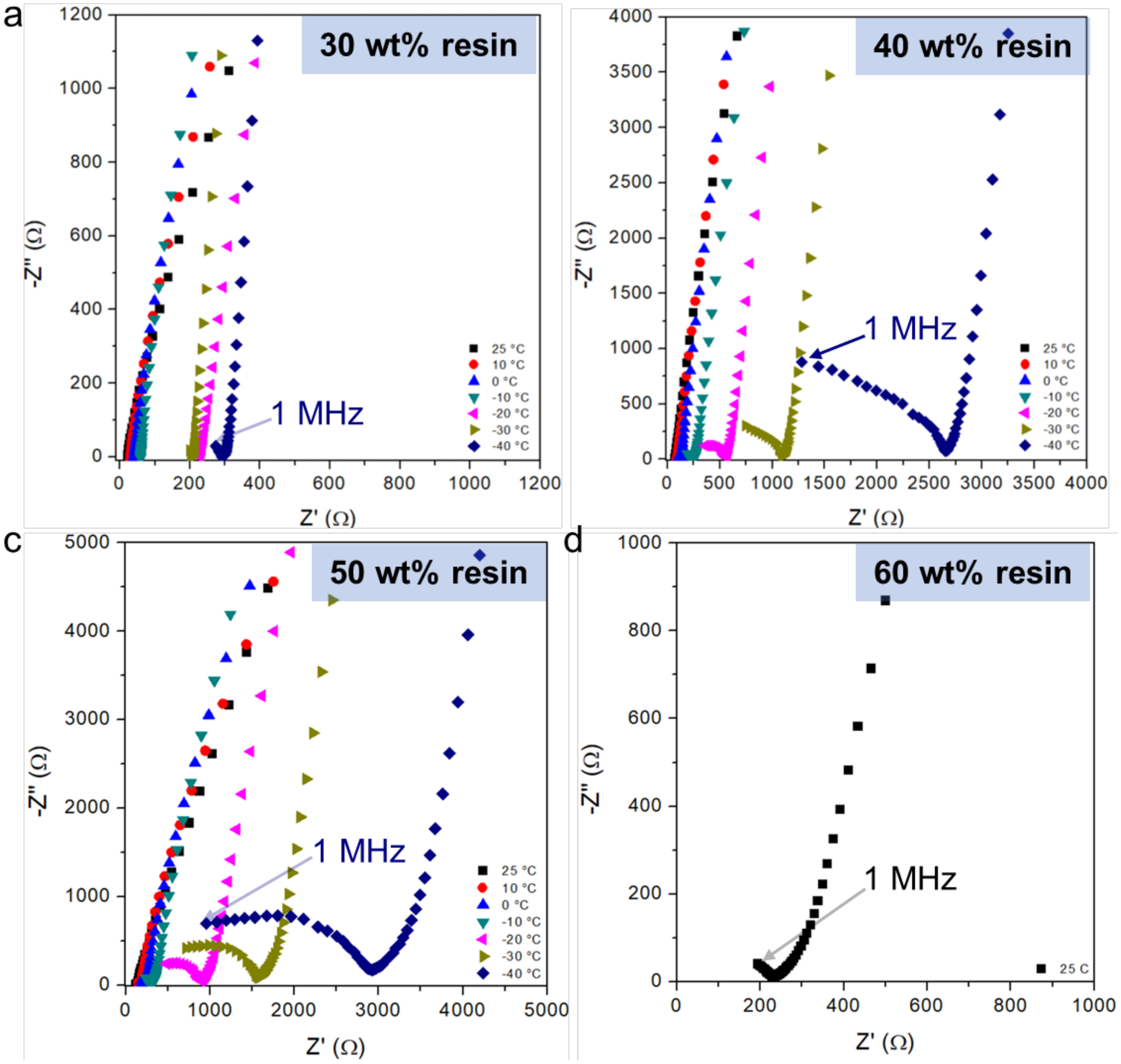

Figure S4. Expanded views of Nyquist plots obtained at different temperatures to calculate ionic conductivity of 1wt% BCP SBEs containing a) 30 wt% resin, b) 40 wt% resin, c) 50 wt% resin, and d) 60 wt% resin.

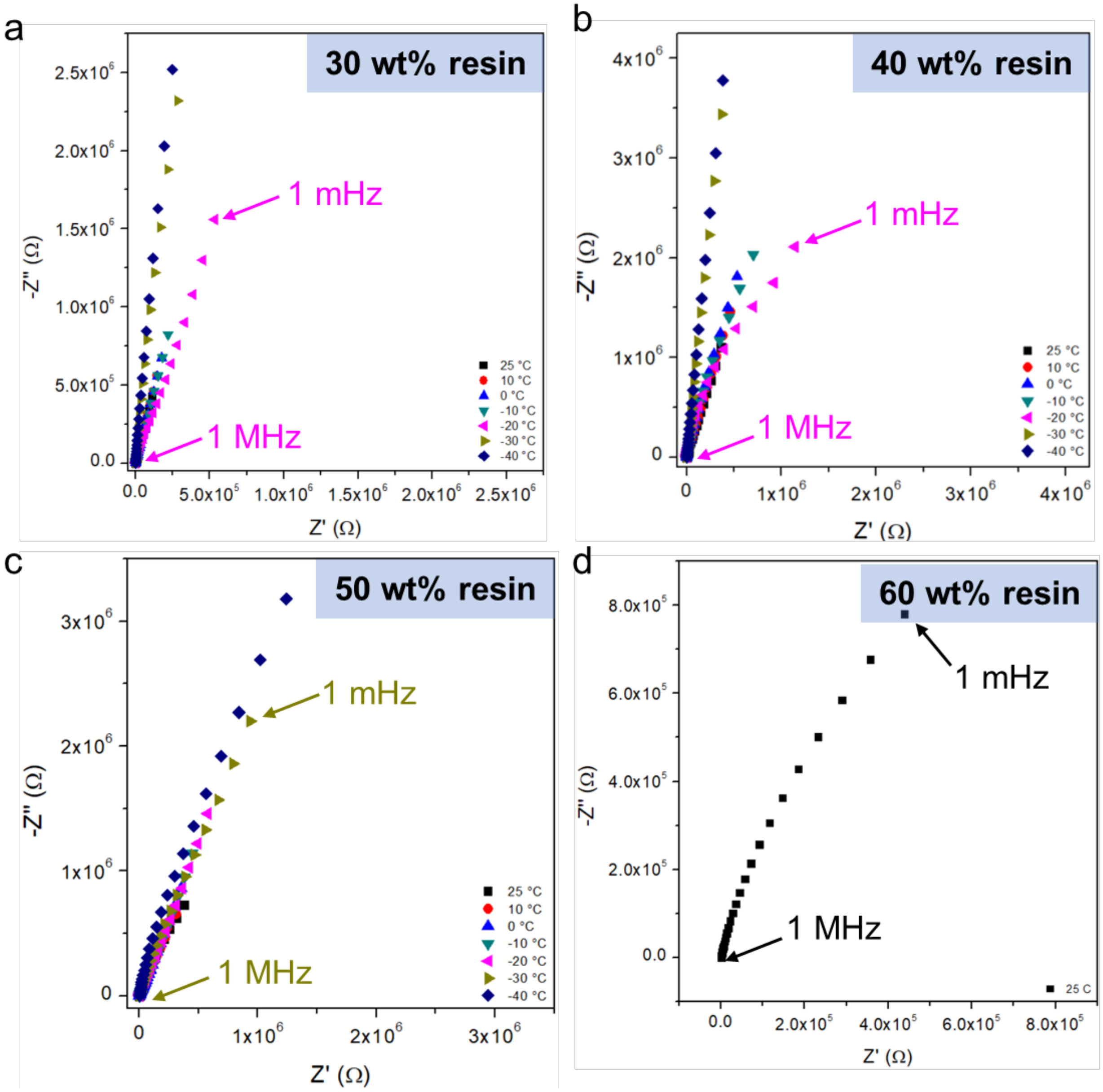

Figure S5. Nyquist plots obtained at different temperatures to calculate ionic conductivity of 1wt% BCP SBEs containing a) 30 wt% resin, b) 40 wt% resin, c) 50 wt% resin, and d) 60 wt% resin.

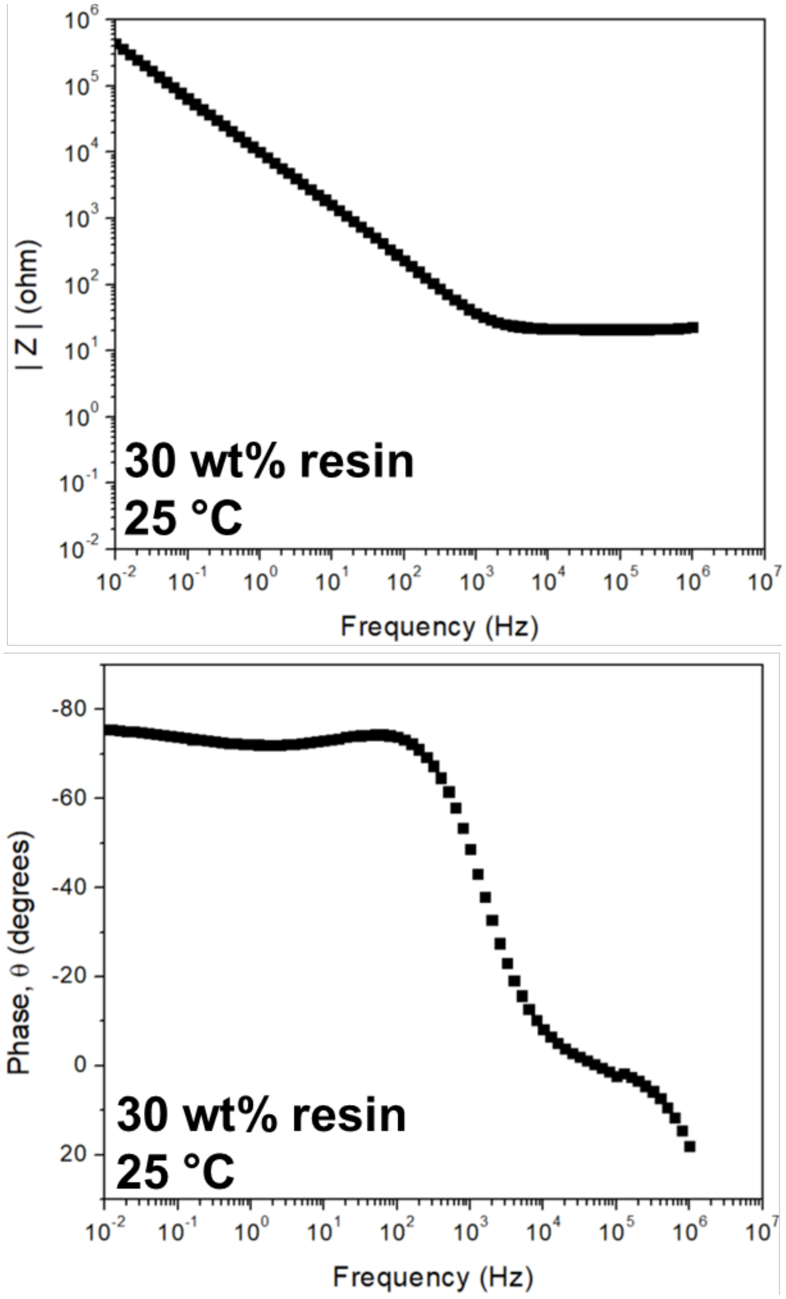

Figure S6. Bode plots of the 1 wt% BCP SBE containing 30 wt% resin at 25 °C.

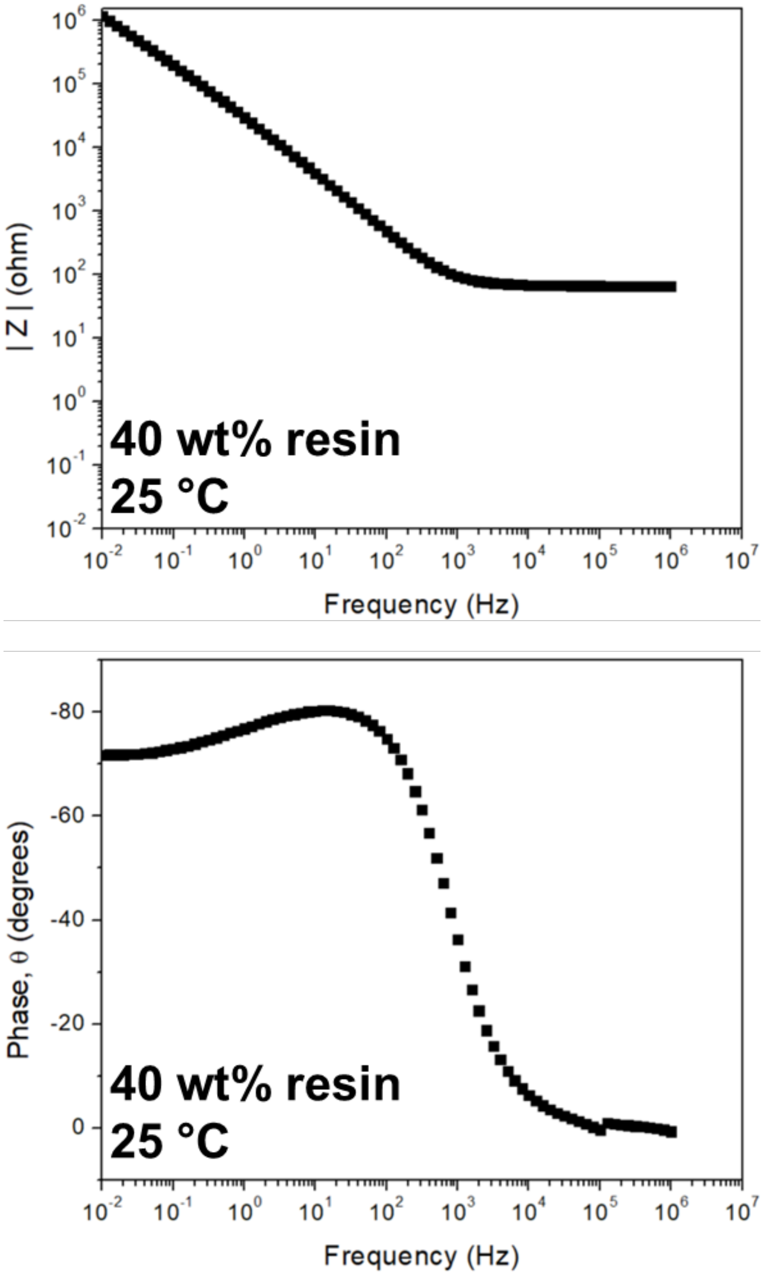

Figure S7. Bode plots of the 1 wt% BCP SBE containing 40 wt% resin at 25 °C.

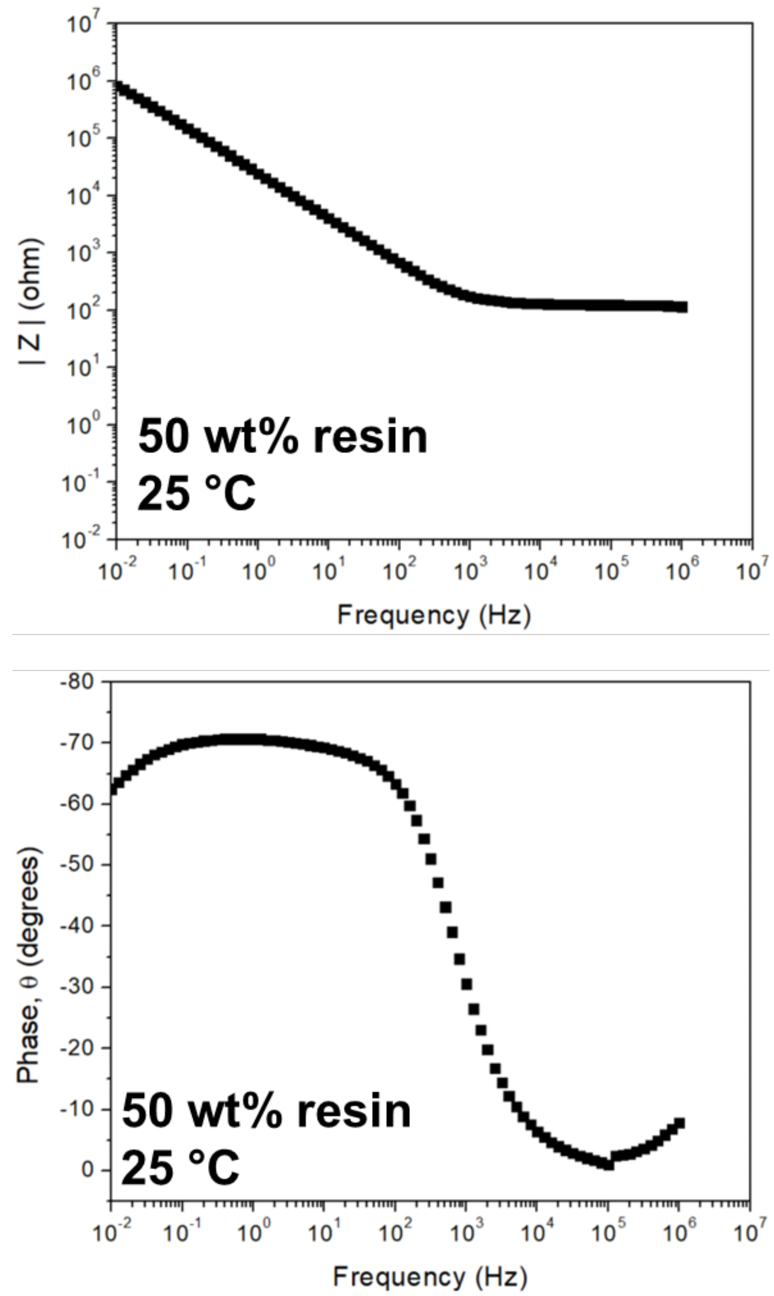

Figure S8. Bode plots of the 1 wt% BCP SBE containing 50 wt% resin at 25 °C.

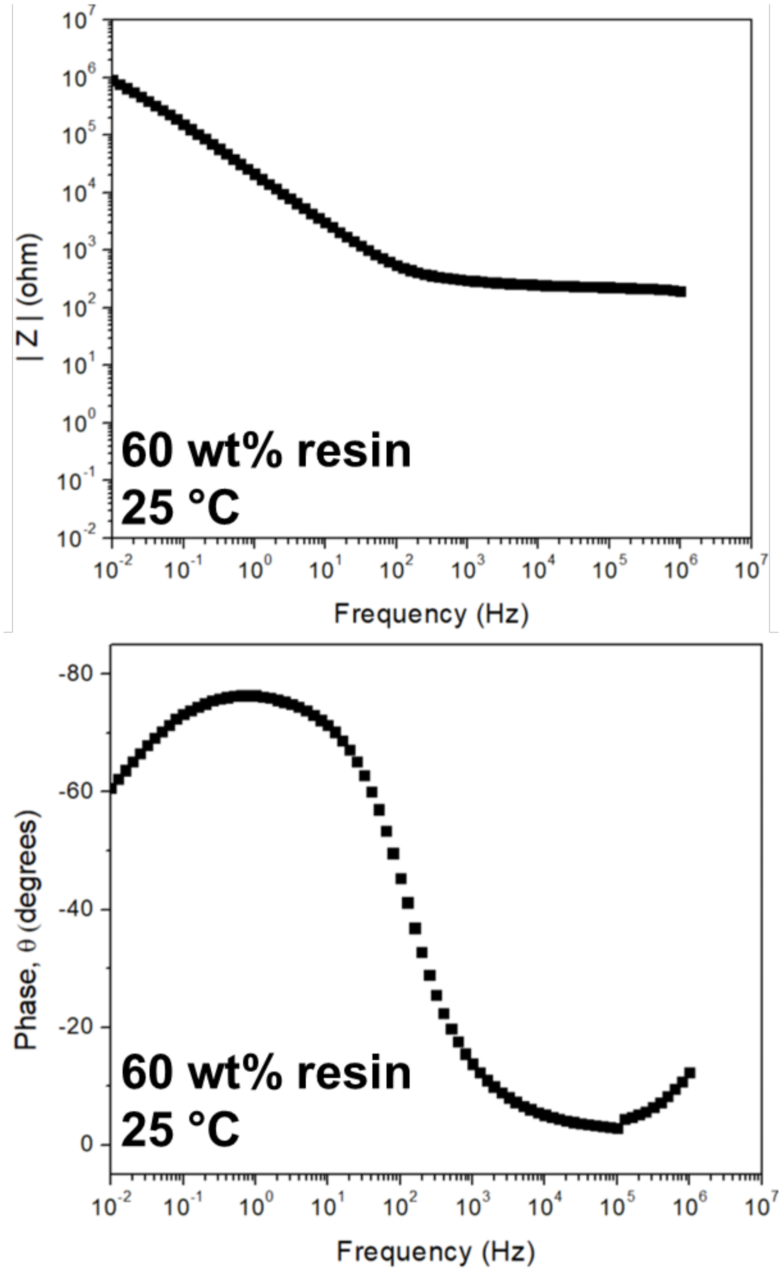

Figure S9. Bode plots of the 1 wt% BCP SBE containing 60 wt% resin at 25 °C.

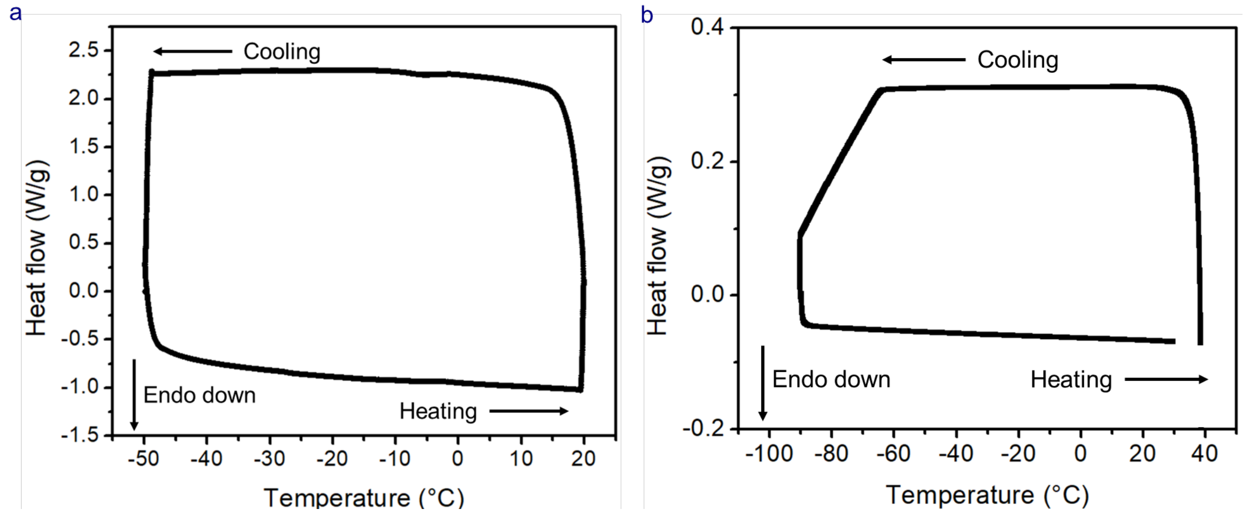

Figure S10. DSC of a) 1wt% BCP SBE with 30 wt% resin and b) neat (100 wt%) liquid electrolyte.

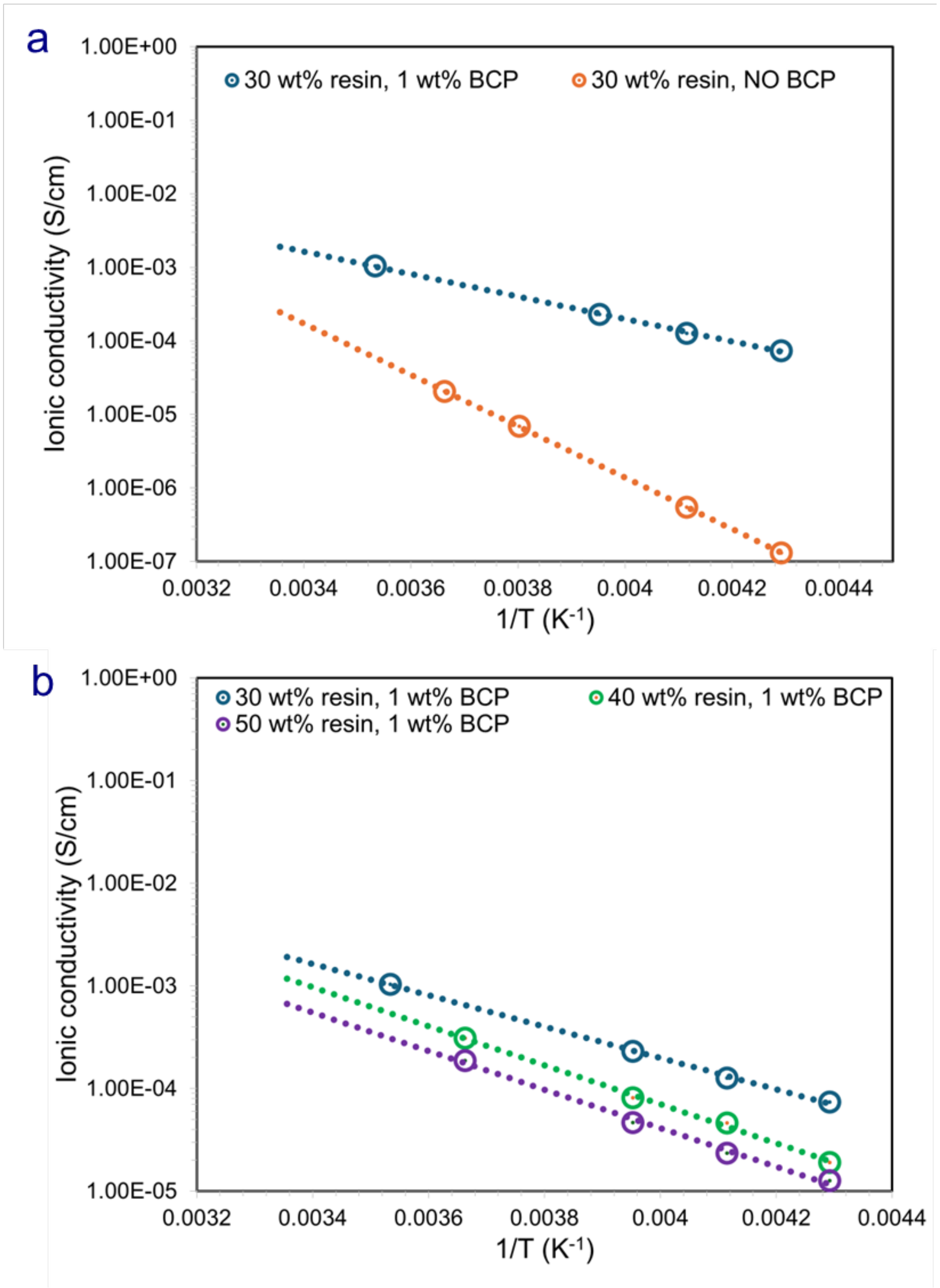

Figure S11. Arrhenius fits over the linear region of the data shown in a) Figure 4a and b) Figure 4b. The Arrhenius dependence of ionic conductivity on T is calculated as

$$\sigma = \sigma_0 e^{-\frac{E}{RT}} \tag{S1}$$

where σ is the ionic conductivity of the electrolyte/SBE, σ_0 is the ionic conductivity of the electrolyte/SBE at 25 °C, E is the activation energy (J/mol), R is the universal gas constant (J/mol K) and T is the absolute temperature (K).[3]

Table S4. Arrhenius fit parameters of the ionic conductivity data for the liquid electrolyte and different SBE compositions plotted in Figures 4a and 4b.

Resin content (wt%)	BCP content (wt%)	Activation energy (kJ/mol K)	R^2
0	0	28.4	0.999
30	0	66.3	0.999
30	1	29.9	0.999
40	1	36.4	0.999
50	1	35.9	0.999

Table S5. Porosity and tortuosity of 1 wt% BCP containing SBEs.

Resin content (wt%)	Effective porosity (ε)	Tortuosity factor (τ)
30	0.560	1.33
40	0.385	2.41
50	0.297	3.00
60	0.149	3.33

Table S6. Porosity and tortuosity of SBEs containing no BCP[2].

Resin content (wt%)	Effective porosity (ε)	Tortuosity factor (τ)
10	0.823	1.80
20	0.569	4.62
30	0.413	17.5
50	0.372	31.9

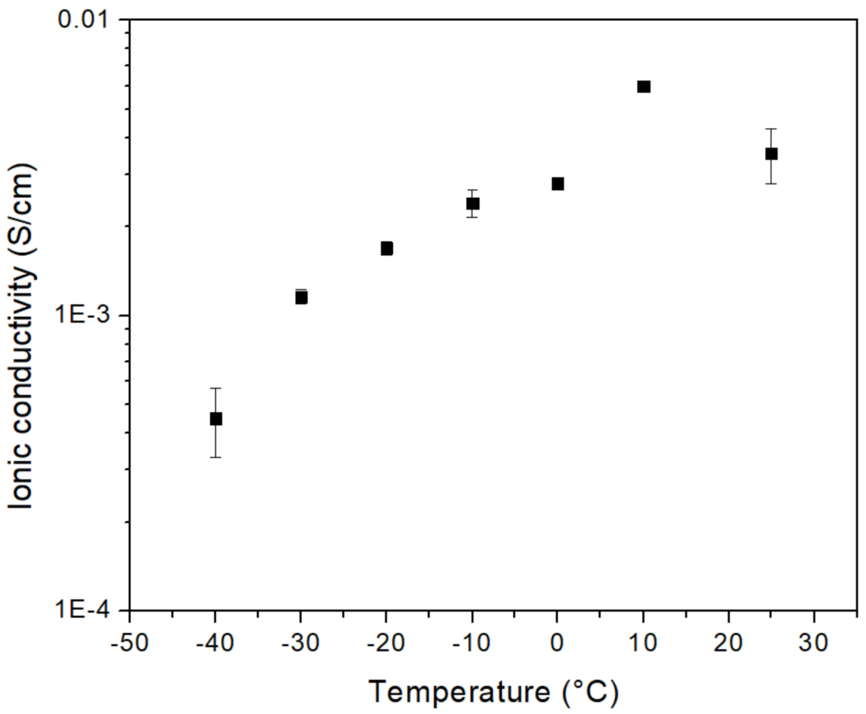

Figure S12. Ionic conductivity of neat (100 wt%) electrolyte as a function of temperature.

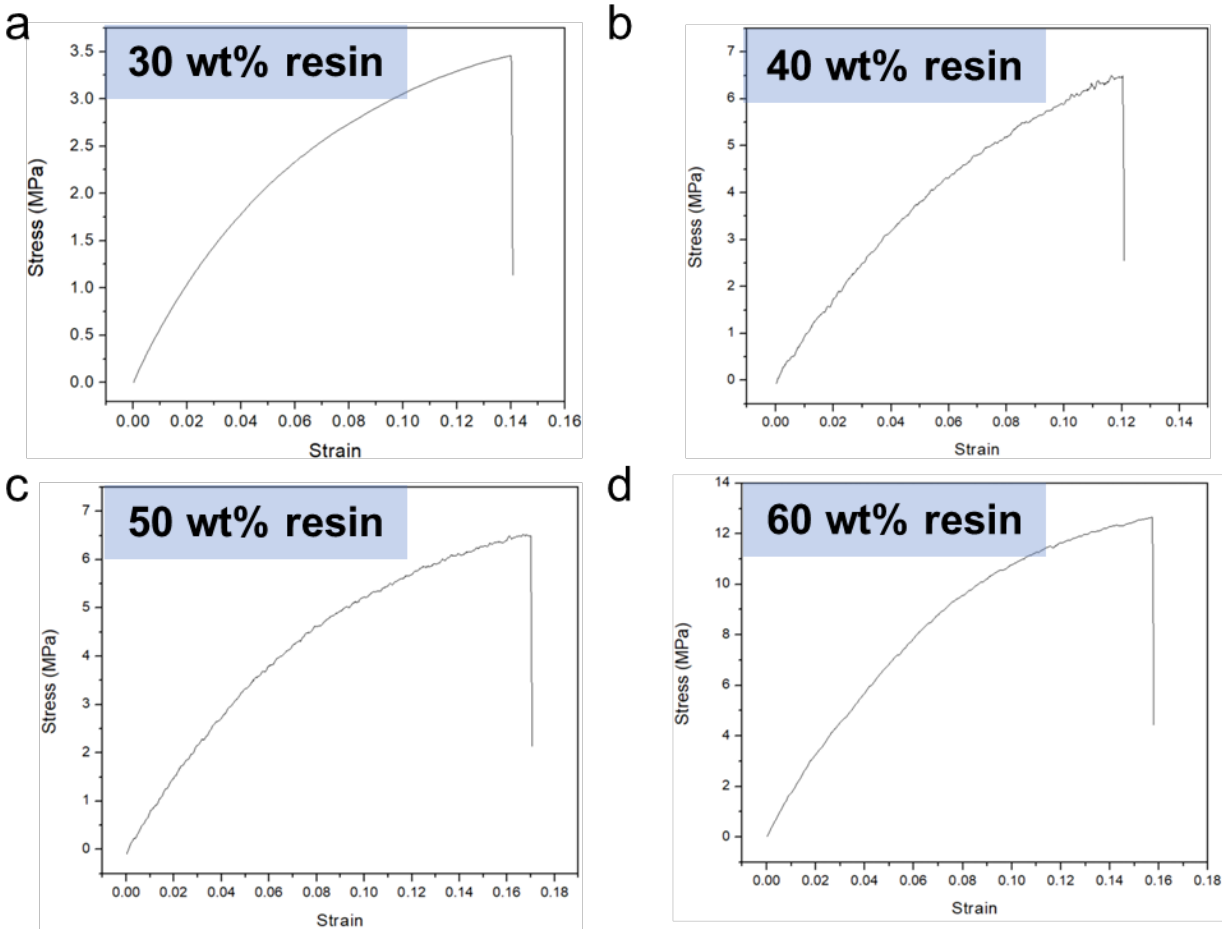

Figure S13. Raw stress-strain curves of 1wt% BCP SBEs containing a) 30 wt% resin, b) 40 wt% resin, c) 50 wt% resin, and d) 60 wt% resin at 25 °C.

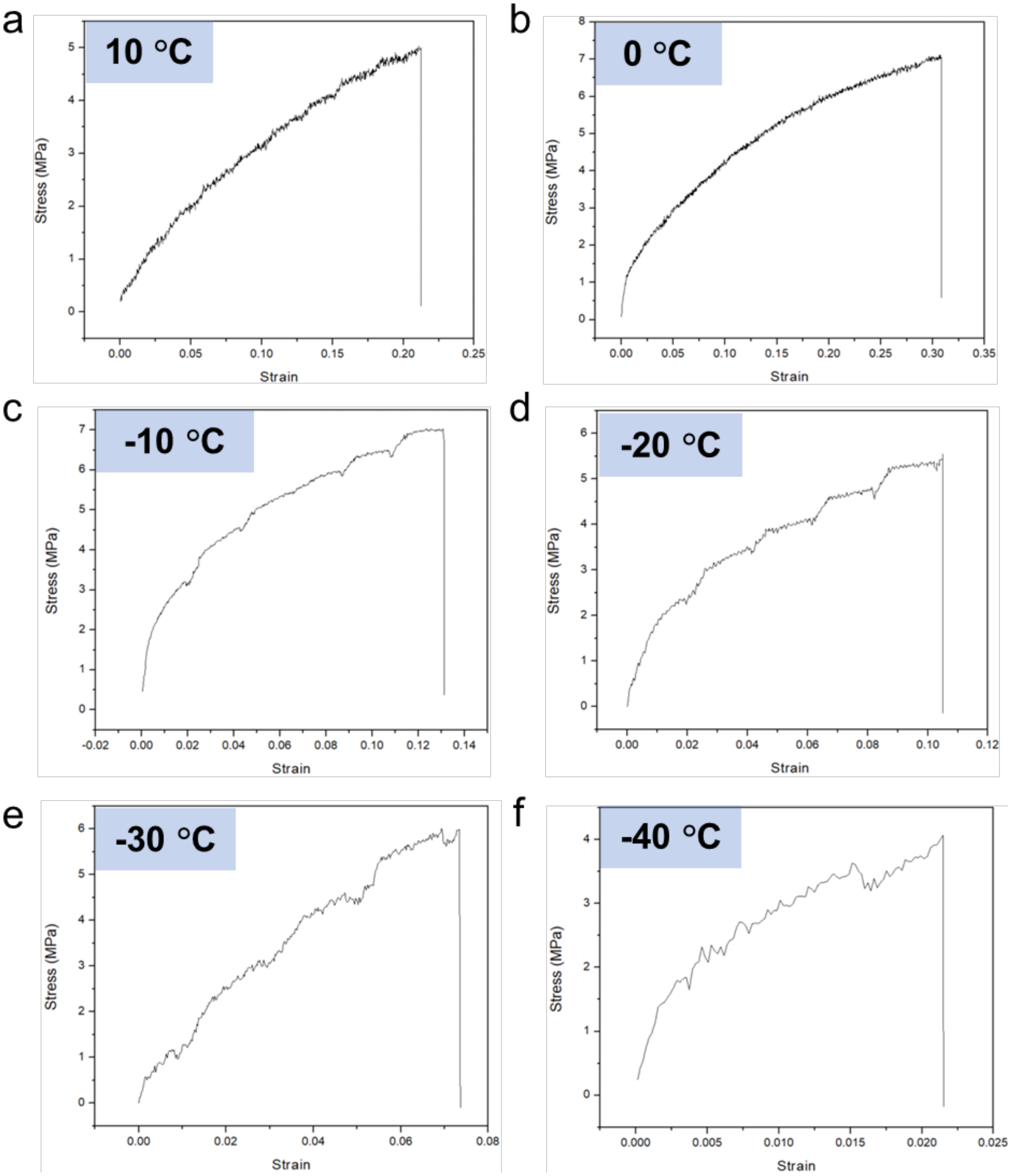

Figure S14. Raw stress-strain curves of 70 wt% resin 1wt% BCP at a) 10 °C, b) 0 °C, c) -10 °C, d) -20 °C, e) -30 °C, and f) -40 °C.

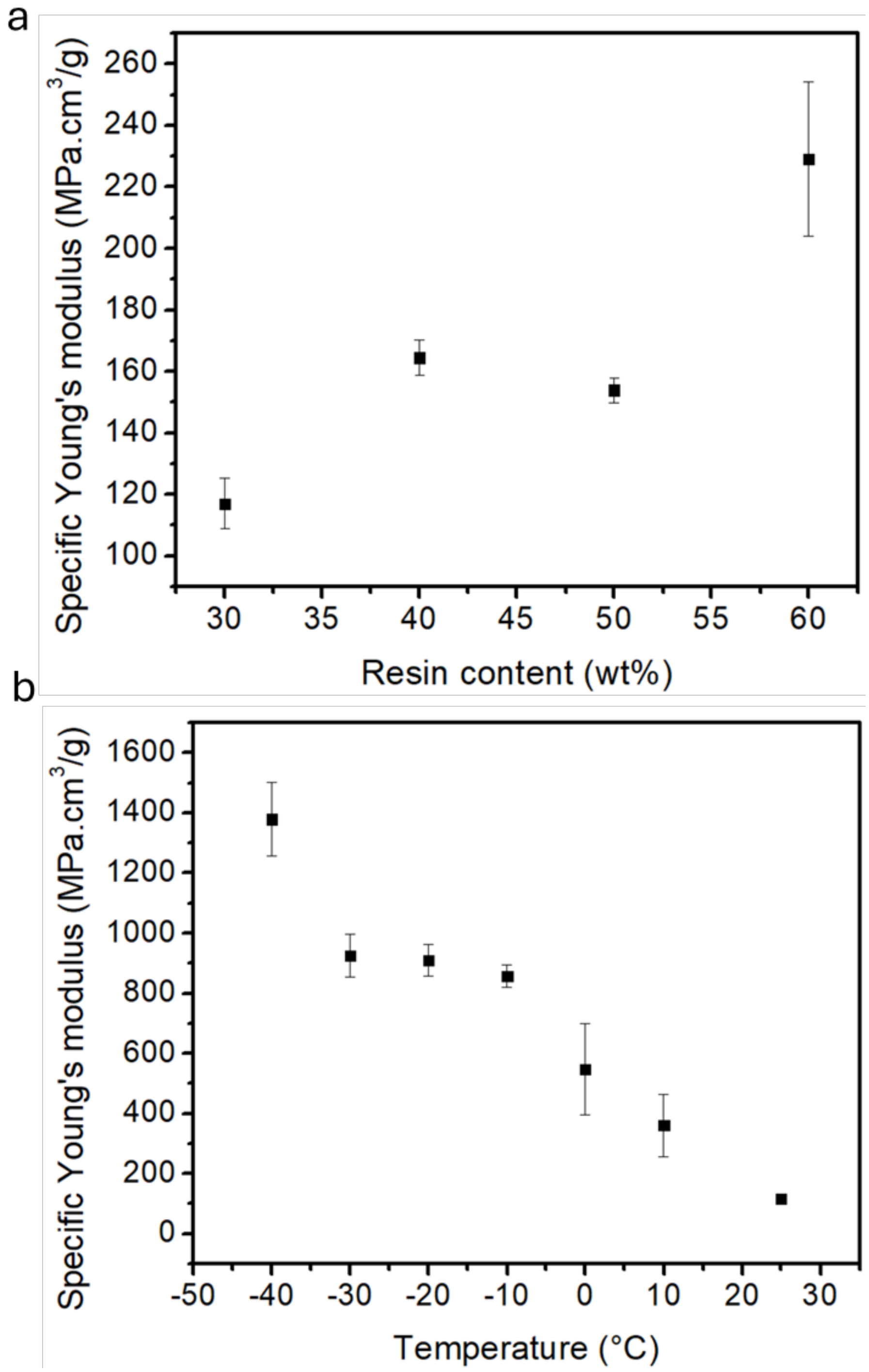

Figure S15. Specific Young's modulus of 1 wt% BCP SBEs (a) as a function of resin content at 25 °C and (b) as a function of temperature at 30 wt% resin SBE.

Table S7. Discharge capacity values for Li/LFP and Li/PTMA-co-GMA cells with 30 wt% resin, 1wt% BCP SBE at low temperatures and different C-rates.

**C-rate**	**Temperature (°C)**	**Discharge capacity (mAh/g)**
0.1	25	145-147
0.2	25	137-139/101-103
0.5	25	120-124/96-97
1	25	103-105/64-67
0.1	10	125-126
0.2	10	111-114/81-83
0.5	10	87-88/69-70
1	10	59-62/25-26
0.1	0	82-90
0.2	0	64-66/72-73
0.5	0	34-35/60-61
1	0	6-9
0.1	-10	67-68
0.2	-10	32-36/68-69
0.5	-10	11-12/51-53
1	-10	3-4
0.1	-20	42-43
0.2	-20	20-22/48-50
0.5	-20	4-5
1	-20	0

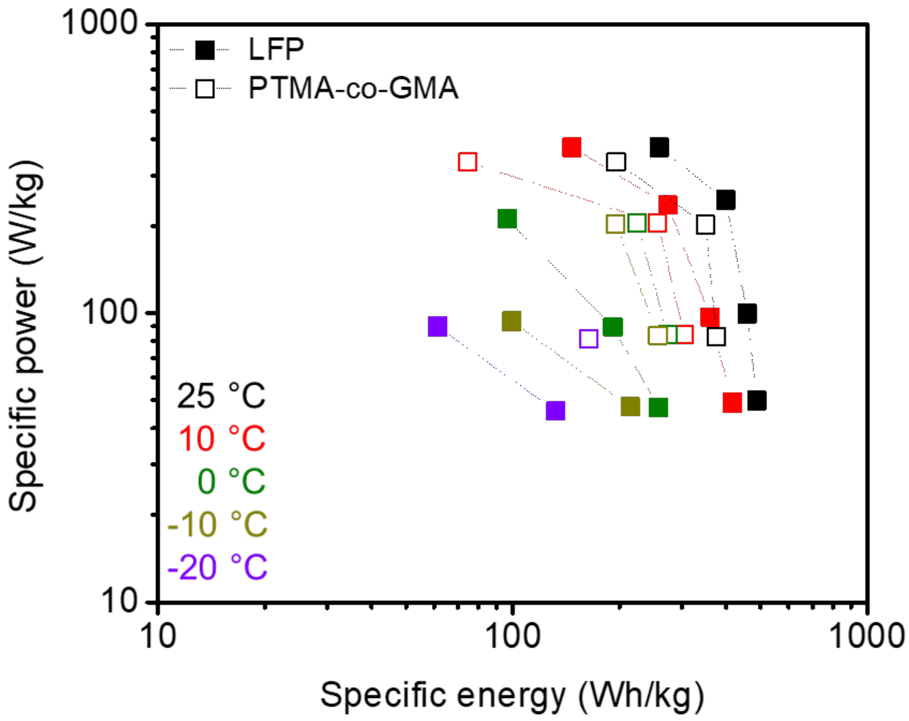

Figure S16. Ragone plots of Li/LFP cells and Li/PTMA-co-GMA cells at 25 °C, 10 °C, 0 °C, -10 °C, and -20 °C, and different C-rates (0.1, 0.2 C, 0.5 C, and 1 C).

Table S8. Specific energy and specific power of Li/LFP and Li/PTMA-co-GMA batteries at different temperatures and C-rates.

C-rate	Temperature (°C)	Specific energy (Wh/kg)	Specific Power (W/kg)
0.1	25	489	49.9
0.2	25	459/376	99.5/82.8
0.5	25	399/350	246/203
1	25	259/196	375/333
0.1	10	416	49.1
0.2	10	361/305	96.9/84.4
0.5	10	274/257	237/205
1	10	147/74.9	375/333
0.1	0	258	47.1
0.2	0	192/274	89.4/84.3
0.5	0	96.7/224	212/204
0.1	-10	215	47.5
0.2	-10	99.6/257	93.9/83.7
0.5	-10	33.5/196	227/204
0.1	-20	132	45.9
0.2	-20	61.5/164	89.8/81.5

References:

(1) Gienger, E. B.; Nguyen, P.-A. T.; Chin, W.; Behler, K. D.; Snyder, J. F.; Wetzel, E. D. Microstructure and multifunctional properties of liquid + polymer bicomponent structural electrolytes: Epoxy gels and porous monoliths. *Journal of Applied Polymer Science* **2015**, *132* (42). DOI: https://doi.org/10.1002/app.42681 (acccessed 2024/12/11).
(2) Deshpande, S.; Vidyaprakash, V.; Oka, S.; Dasari, S. S.; Liu, K.-W.; Wang, C.; Lutkenhaus, J. L.; Green, M. J. Correction to "Low-Temperature Structural Battery Electrolytes Produced by Polymerization-Induced Phase Separation". *ACS Applied Polymer Materials* **2024**, *6* (17), 11066-11069. DOI: 10.1021/acsapm.4c02519.
(3) Petrowsky, M.; Frech, R. Temperature Dependence of Ion Transport: The Compensated Arrhenius Equation. *The Journal of Physical Chemistry B* **2009**, *113* (17), 5996-6000. DOI: 10.1021/jp810095g.

:
